# Genomic resources for wild populations of the house mouse, *Mus musculus* and its close relative *Mus spretus*

**DOI:** 10.1038/sdata.2016.75

**Published:** 2016-09-13

**Authors:** Bettina Harr, Emre Karakoc, Rafik Neme, Meike Teschke, Christine Pfeifle, Željka Pezer, Hiba Babiker, Miriam Linnenbrink, Inka Montero, Rick Scavetta, Mohammad Reza Abai, Marta Puente Molins, Mathias Schlegel, Rainer G. Ulrich, Janine Altmüller, Marek Franitza, Anna Büntge, Sven Künzel, Diethard Tautz

**Affiliations:** 1 Max-Planck Institute for Evolutionary Biology, August-Thienemanstrasse 2, 24306 Plön, Germany; 2 Department of Medical Entomology and Vector Control, School of Public Health, Tehran University of Medical Sciences, Tehran 1417613151, Iran; 3 Laboratorio de Anatomía Animal, Departamento de Biología Animal, Facultad de Ciencias, Universidad de Vigo, 36200 Vigo, Spain; 4 Friedrich-Loeffler-Institut, Federal Research Institute for Animal Health, Institute for Novel and Emerging Infectious Diseases, Südufer 10, 17493 Greifswald-Insel Riems, Germany; 5 Cologne Center for Genomics (CCG), University of Cologne, Weyertal 115b, 50931 Cologne, Germany; 6 Institute of Human Genetics, Universitätsklinik Köln, Kerpener Str. 34, 50931 Köln, Germany; 7 Cologne Excellence Cluster on Cellular Stress Responses in Aging-Associated Diseases (CECAD), University of Cologne, Joseph-Stelzmann-Str. 26, 50931 Cologne, Germany

**Keywords:** Population genetics, Mouse, Molecular ecology, DNA sequencing, RNA sequencing

## Abstract

Wild populations of the house mouse (*Mus musculus*) represent the raw genetic material for the classical inbred strains in biomedical research and are a major model system for evolutionary biology. We provide whole genome sequencing data of individuals representing natural populations of *M. m. domesticus* (24 individuals from 3 populations), *M. m. helgolandicus* (3 individuals), *M. m. musculus* (22 individuals from 3 populations) and *M. spretus* (8 individuals from one population). We use a single pipeline to map and call variants for these individuals and also include 10 additional individuals of *M. m. castaneus* for which genomic data are publically available. In addition, RNAseq data were obtained from 10 tissues of up to eight adult individuals from each of the three *M. m. domesticus* populations for which genomic data were collected. Data and analyses are presented via tracks viewable in the UCSC or IGV genome browsers. We also provide information on available outbred stocks and instructions on how to keep them in the laboratory.

## Background & Summary

The house mouse (*Mus musculus*) has a long-standing history as a model system in genetics and biomedical research, with many classical inbred strains available for purchase worldwide. By comparing the genetic make-up of classical inbred strains with those of mice collected in the wild, it became clear that classical inbred strains represent complex genomic mixtures with contributions from different subspecies and species of *Mus*^1–3^. While some of this genomic mixture stems from captive breeding of mice from different parts of the world during the early establishment of inbred strains, admixture^4,5^ and introgression of genomic material across subspecies and species^6–9^ also occurs in the wild and is thus likely to contribute to the genomic complexity observed in inbred strains. Classical inbred strains were found to exhibit a much reduced amount of genetic variation compared to their wild mice ancestors^10^. For example, all classical inbred strains share a single mitochondrial lineage derived from *M. m. domesticus*^11^, indicating that they all descend from the same female lineage of the wild ancestor.

An appreciation of the genetic diversity found in wild mice came with the advent of molecular mapping techniques that required crosses between lines with informative polymorphisms^12,13^. This has led to renewed interest in studying the evolutionary history of natural house mouse populations worldwide, with a main focus on clarifying its taxonomy and catalogue genetic variations found in the wild^14–16^. Currently, three major lineages of *Mus musculus*, classified as subspecies, are distinguished: the western house mouse *Mus musculus domesticus*, the eastern house mouse *Mus musculus musculus* and the southeast-Asian house mouse *Mus musculus castaneu*s. All three lineages have their origin in Southern Asia and diverged roughly 0.5 million years ago, but still share haplotypes and appear to exchange genomic material^6,8,9^. Hybrid zones have been detected at areas of secondary contact between the subspecies^17–19^ and these serve for tracing genes involved in hybrid incompatibility^20–22^ as well as quantitative trait mapping^23^.

During the past 10,000 years house mice have developed commensalism with humans, which allowed them to a spread across the world. Among the recognized subspecies, *M. m. domesticus* seems to be the most successful colonizer of new continents during the past few hundred years^12,13^, partly revealing historical shipping routes of humans^24^, and has repeatedly colonized small islands^25^. One such recent island colonization occurred about 400 years ago and resulted in the naming of a new subspecies, i.e., *M. m. helgolandicus*^26^. Because of its molecular proximity to *M. m. domesticus*, we treat the *M. musculus* population from Heligoland, a small German archipelago in the North Sea, as a member of the subspecies *M. m. domesticus* in some further analyses.

One of the closest relatives to the *Mus musculus* subspecies complex is the Algerian mouse *Mus spretus*^27^, with populations inhabiting areas around the western Mediterranean Sea. With a divergence time of roughly 2 million years^28,29^ the *Mus spretus* lineage serves as an ideal outgroup to *Mus musculus*. Although viable offspring can be produced from crosses between *Mus musculus* and *Mus spretus* in the laboratory, it is morphologically and behaviorally rather distinct, justifying its status as separate species. Nevertheless, some exchange of genomic regions is still possible between these species in the wild^7,9,30^. A few individuals of *Mus spretus* are also represented among classical inbred strains^31^.

The unique combination of genetic and molecular knowledge derived from the classical inbred strains and the profound knowledge of the evolutionary history of wild mouse populations make *Mus musculus* a prime model for the study of evolution and molecular biology of natural populations^12,13,32^. We have previously used the *Mus musculus* model system to analyze patterns of positive and negative selection in the genome^8,33–37^, hybrid sterility^21,22^, evolution of copy number variation^38^, mapping of craniofacial traits^23^ and the composition and turnover of the microbiota^39–41^.

**Figure 1:**
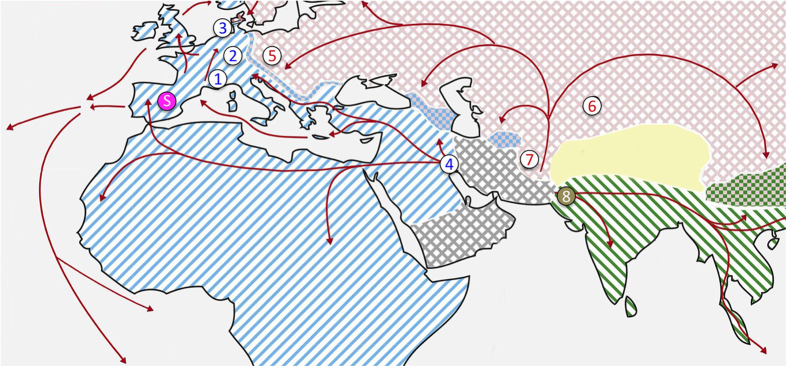
Geographic location of *Mus musculus* (1–8) and *Mus spretus* (S) samples. The map is modified from refs 3,13. The blue area depicts *M. m. domesticus* territory (includes *M. m. helgolandicus* (3), because of its close molecular proximity to *M. m. domesticus*), the red area depicts *M. m. musculus* territory, and the green area depicts *M. m. castaneus* territory. *Mus spretus* co-occurs with *M. m. domesticus* in Spain. The grey area harbors further lineages and possible additional subspecies^16^. Red arrows symbolize possible migration routes, mostly in post-glacial times during the spread of agriculture. Locations (year caught): 1, Massif Central/France (2005); 2, Cologne-Bonn/Germany (2006); 3, Heligoland/Germany (2012); 4, Ahvaz/Iran (2006); 5, Studenec/Czech Republic (2003); 6, Almaty/Kazakhstan (2002); 7, Afghanistan (2012); 8, Himachal Pradesh/India (2003); S, Madrid/Spain (2004).

Here we describe the genomic resources that we have generated using mice collected in the wild over the past 10 years. These data can serve as a basis for in depth studies at a population level and to inform biomedical research projects on natural polymorphisms that are present in inbred strains. We provide information on a) genomic data for a total of nine populations (Fig. 1), covering the three major house mouse subspecies and one outgroup, b) tissue-specific RNAseq data from three *M. m. domesticus* populations, and c) details on animal husbandry of wild house mice in a Supplementary File and d) some general analyses and browser tracks for visualization. The genomic dataset is summarized using basic descriptive statistics, such as F_ST_ (ref. 42), π (nucleotide diversity^43^) and Tajima’s D^44^ as measures of population differentiation and selection, as well as a description of copy-number variation based on sequencing read depth. The RNAseq data are summarized as normalized RNAseq read coverage for each base pair of the genome. All statistics are made available as genome browser tracks (bed and bigWig files) to allow close visual inspection of any genomic region of interest in the UCSC browser^45^ or other visualization software such as IGV^46,47^.

## Methods

### Sampling procedure and sampling locations

The location of populations used for re-sequencing in this study are depicted in Fig. 1. They include three *M. m. domesticu*s populations from Western Europe and Iran, the island subspecies *M. m. helgolandicus*, three *M. m. musculus* populations from the Czech Republic, Kazakhstan and Afghanistan and a *M. spretus* population from Spain. Populations were sampled between 2003 and 2012. The DNA samples used for genome sequencing were obtained either directly from wild caught animals, or from the first or second generation of out-breeding in our animal facility, i.e., they are expected to represent full wild type variation. Some aspects of the genome sequences obtained from the three *M. m. domesticu*s populations, as well as the island subspecies *M. m. helgolandicu*s, have previously been described^26,38^.

House mice form naturally extended family groups at a given location including breeding among relatives^48,49^. To obtain an unbiased population sample from a given region ideally requires sampling mice in a way to avoid catching related animals. Therefore, we aimed to collect only a single mouse per trapping location (or, for the purpose of setting up breeding colonies, one male and one female per location) and selected the next trapping location 500 m to 1 km apart. The whole area sampled ideally comprises a diameter of about 50 km. However, depending on the local conditions, following this sampling regime precisely was not always possible. Moreover, some samples were provided by collaborators who have only recorded the general area for trapping, but not the exact location (e.g., the mice from Kazakhstan). Other trapping locations had regional limitations. For example, the island of Heligoland is only 1.7 km^2^ in size, or the military Camp Marmal, Mazar-e-Sharif (Afghanistan) is only 8.7 km^2^ in size. In both cases, mice were collected in different localities on the island or military base respectively^50^. In Supplementary Table 1 we provide exact location information, as much as it is available for all animals involved in the study, as either directly having been sequenced, or as having been parent to one of the animal facility-born offspring of wild mice (see below).

To complement the genomic resources generated in our laboratory, we also re-analyzed previously published genome sequencing data from *M. m. castaneus* that were collected in the northwest Indian state of Himachal Pradesh^51^.

Mice were either caught in snap traps, or live traps (‘Mäusewippfalle’ No. 3451002, Firma Ehlert & Partner, 53859 Niederkassel, Germany) or were found dead after rodenticide-based pest management. Cervical dislocation was used to sacrifice mice caught in live traps in the field. Transportation of live mice to the animal facility, maintenance and handling were conducted in accordance with German animal welfare law (Tierschutzgesetz) and FELASA guidelines. Permits for keeping mice were obtained from the local veterinary office ‘Veterinäramt Kreis Plön’ (permit number: 1401-144/PLÖ-004697).

### *M. m. domesticus* breeding scheme

For the *M. m. domesticus* populations our aim was to generate an RNAseq dataset from the same individuals for which we generated the DNAseq data. In order to standardize mice to the same sex, age and environmental conditions, we did not use wild caught mice but bred wild caught mice for one to two generations in our animal facility. Supplementary Table 2 shows the breeding scheme for the *M. m. domesticus* mice in the study. In one case, we caught a pregnant female in the wild and used its male offspring born in the facility for DNA and RNA sequencing. For the Iranian mice, we included two wild caught individuals in the DNAseq study (AH15 and AH23), for which we did not generate RNAseq data. For two additional Iranian individuals in the DNA sequencing study, tissue samples were lost and thus no corresponding RNAseq data exist. To compensate for this, we included four individuals representing male offspring from male AH15 and male AH23 respectively (both individuals are part of the DNAseq dataset; see Table 1 (available online only)). Two male offspring from each of these two males are represented as biological replicates in the RNAseq dataset.

### Procedures for wild mouse handling

We established wild-derived outbred populations for *M. m. domesticus* (France, Germany) and *M. m. musculus* (Kazakhstan and Czech Republic). For the first 11–14 generations live mice obtained from the wild were set up in a cyclical breeding scheme aiming at maintaining maximum genetic variability over time. They were then partly refreshed with newly collected mice from the same original area and a HAN rotational breeding scheme^52^ was established. Individuals from each of the 4 outbred populations are maintained at the Max-Planck Institute in Plön, Germany, and are available from the authors upon request.

Wild mice are considerably more agile than classical inbred strain mice. Environmental enrichment is necessary and strongly reduces agitated stereotype behavior in wild mice kept under laboratory conditions. Standard mouse chow is provided *ad libitum* (e.g., Altromin 1,324 from ALTROMIN, 32,791 Lage, Germany). Further details on mouse handling and breeding are provided in Supplementary Material Text 1.

### Molecular methods

#### DNA and RNA extraction

DNA was extracted from liver, spleen, or ear samples using salt extraction^53^ or DNeasy kits (Qiagen, Hilden, Germany). RNA was extracted only for *M. m. domesticus* mice from Germany (8 individuals), France (8 individuals) and Iran (8 individuals) using mostly the same individuals, which are included in the whole genome sequencing study (see details above and Supplementary Table 2).

Mice designated for RNA extraction were housed alone after weaning and were routinely visually inspected for health and vigor. Only male mice were included in the study, sacrificed at 12 weeks. All mice were fed standard mouse chow *ad libitum* (Altromin 1,324 from ALTROMIN, 32,791 Lage, Germany). Mice were sacrificed by CO_2_ asphyxiation. The coat was sprayed with 75% EtOH to reduce loose hair contaminating the organs during dissection of the animal. Organs were extracted in a specific order to improve comparability. Organs were shock frozen in liquid nitrogen and stored at −80° until RNA was extracted.

The Trizol reagent (Life Technologies, Carlsbad, California, USA) was used according to manufacturers instructions to extract RNA from each organ (Table 2 (available online only)). With the exception of the liver, for which we used only right and left medial lobe, whole organs were processed to minimize heterogeneity of the sample. The extracted RNA was quantified on a Nanodrop and analyzed for integrity on the Agilent Bioanalyzer.

#### DNA sequencing library preparation

All populations apart of the Afghanistan population were sequenced using the same protocol (see below) in the following batches: Batch 1 included the *M. m. domesticus* populations from France, Germany and Iran (locations 1, 2 and 4 in Fig. 1). Batch 2 included the 3 mice from Heligoland (location 3 in Fig. 1). Batch 3 included the *M. m. musculus* populations from the Czech Republic and Kazakhstan (locations 5 and 6 in Fig. 1) as well as the *M. spretus* population from Spain (location S in Fig. 1). Batch 4 included the *M. m. musculus* mice from the Afghanistan population (location 7 in Fig. 1), which were sequenced using a more recent Illumina technology (see below).

For whole genome sequencing of batch 1–3 we fragmented 1 μg of DNA of each individual using the 250 bp sonication protocol (Bioruptor, Diagenode, Liège, Belgium). The fragments were end-repaired and adaptor-ligated, including incorporation of sample index barcodes. The products were then purified and amplified (10 PCR cycles) to create the final libraries. The TruSeq DNA LT Sample Prep Kit v2 was used for all steps. After validation (Agilent 2,200 TapeStation), all libraries were quantified using the Peqlab KAPA Library Quantification Kit and the Applied Biosystems 7900HT Sequence Detection System. One library was loaded on two lanes of a Hiseq2000 sequencer and sequenced with a 2×100 bp v3 protocol.

The DNA quality of the Afghanistan mice (batch 4) proved problematic. Therefore, to recover high molecular weight genomic DNA, we ran a 0.7% agarose gel over night and extracted the genomic DNA from the gel using the Zymoclean Large Fragment DNA Recovery Kit (Zymo Research Europe, Freiburg im Breisgau, Germany). For whole genome sequencing we used the Nextera DNA library Prep Kit (Illumina) following manufacturer’s instructions and 50 ng of genomic DNA as starting material. Each sample was run on Agilent Bioanalyzer using the Agilent DNA7500 kit to verify that the fragment sizes were in the 500 bp range. To calculate the final concentration for the sequencing run, the samples were measured with the Quant-iT dsDNA BR Assay Kit on a Nanodrop 3,300 fluorometer. The samples were paired-end sequenced (76 bp) independently on a single flow cell on a NextSeq 500 using the NextSeq 500 High Output v2 150 cycles chemistry.

#### RNA sequencing library preparation

We used the NEBNext Ultra RNA Library Prep Kit for Illumina with the Poly(A) mRNA Magnetic Isolation module to generate the RNAseq libraries. We used a fragmentation time of 15 min (yielding ~180 bp fragments) and 15 PCR cycles for library enrichment. After validation (Agilent 2,200 TapeStation), all libraries were quantified using the Peqlab KAPA Library Quantification Kit and the Applied Biosystems 7900HT Sequence Detection System. Samples were pooled such that we generated about 12 million paired reads/sample, which were loaded on one lane each of a Hiseq2000 sequencer and sequenced with a 2×100 bp v3 protocol.

### Data analysis

#### Mapping genomic reads and Single Nucleotide Polymorphism (SNP) calling

**Figure 2:**
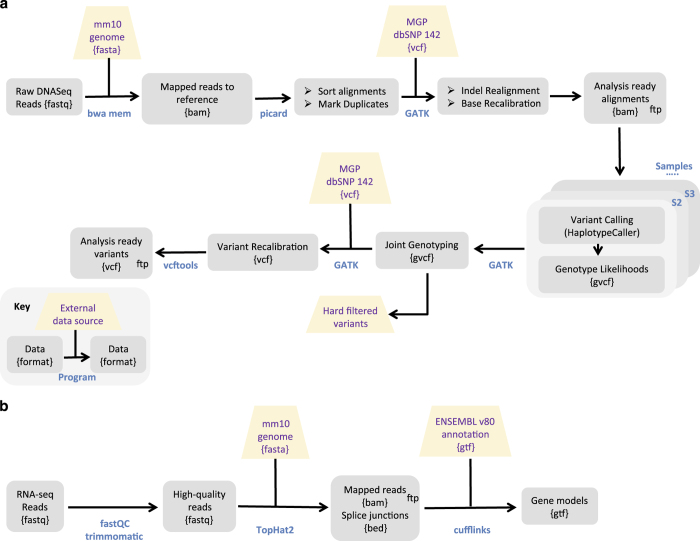
Overview of mapping pipeline for genomic (**a**) and transcriptomic (**b**) reads. See Supplementary Material Text 2 for full details. Analysis steps for which files are provided are marked with ‘ftp’.

All sequencing reads (including those of the 10 previously published *M. m. castaneus*^51^ genomes) were processed according to a single standardized pipeline, which is outlined in Fig. 2a and described in detail (including commands) in Supplementary Material Text 2. In brief, reads were mapped against the mouse *mm10* genome reference sequence^54^ (http://www.ncbi.nlm.nih.gov/projects/genome/assembly/grc/mouse/) using bwa-mem^55^. The Picard tools software suite (http://broadinstitute.github.io/picard/) was used for sorting, marking and removing duplicates. Raw SNP and indel calls were obtained from the alignment files following precisely the GATK^56^ ‘Best Practice’ instructions on joint genotyping of all samples together. The raw .vcf files were subjected to the GATK VSQR SNP filtering step, which uses known variants as training data to predict whether a new variant is likely a true positive, or a false positive. As training data we used the file ‘mgp.v5.merged.snps_all.dbSNP142.vcf’ downloaded from ftp://ftp-mouse.sanger.ac.uk/current_snps/^57^ which was filtered for ‘PASS’ SNPs. In addition, we used very stringent hard filtering criteria on our own dataset, and included these SNPs as training sets as well (see details in Supplementary Material Text 2). Due to an absence of a reliable indel reference dataset we did not generate VSQR calls for indels. Thus, all indels called in the .vcf file should be considered ‘raw’.

On our ftp website we provide a .vcf file with all SNPs and indels where we flag all SNPs within the 90% VSQR tranche as ‘PASS’ SNPs (this means that we accept all variants until we reach 90% of our known ‘truth’ set). This is a rather conservative filter on SNP quality, but users are free to perform their own filtering using their own training set and parameters for filtering on the raw .vcf file.

#### Transcriptome sequencing of M. m. domesticus populations

Transcriptome sequencing reads were processed according to the pipeline depicted in Fig. 2b. In short, they were trimmed according to quality values using *Trimmomatic*^58^, removing bases below Q20 and maintaining an average read quality above Q25. Pairs with one read below the quality thresholds were removed from the analyses. Quality-checked (QC) reads were mapped against the mouse *mm10* genome reference using *TopHat2* (ref. 59) and using the default settings for paired-end samples. The output alignments were sorted and indexed with *samtools*^60^. The sorted alignments were assigned to the version 82 of Ensemble Mouse gtf annotation^61^ using *featureCounts* from the *subreads* suite^62^ in paired-end (-p) exon mode (default). The gtf file contained only linear complete chromosomes (no scaffolds, no mitochondria). The unmapped pairs were counted with *featureCounts* from the ‘<unmapped.bam>’ file *TopHat2* generates. Percentages are reported relative to the total number of QC reads. On average (across all tissues and all samples) 93% of the total number of QC reads could be mapped to the *mm10* genome (range 86.5–96%, Table 2 (available online only), Supplementary Table 3). This number also includes spliced reads, which *Tophat2* detects. This number dropped to 57% (range 33–66%) for reads uniquely mapping to ENS 82 annotated features (i.e., exons, Supplementary Table 3).

#### Copy number variation (CNV) analyses

We used the sequencing read depth approach implemented in the *CNVnator* software^63^ to predict CNV calls relative to the mouse *mm10* reference assembly. We have previously experimentally confirmed that this is a reliable approach^38^. Optimal bin size for each individual was chosen such that the ratio of the average read depth signal to its standard deviation was between 4 and 5. Bin size ranged from 100–1,500 bp and was inversely proportional to genome coverage. Only linear complete chromosomes were considered. Calls intersecting annotated gaps in the reference genome were not considered. The CNV detection statistics are provided in Table 3 (available online only). Haploid copy numbers for each detected CNV, either per population or per individual, are included in the bed files for the UCSC browser tracks (available at the ftp site).

#### Visualization of data within and between populations

For the genomic data we set up UCSC^45^ genome browser tracks for 10 kb windows of nucleotide diversity π, Tajima´s D and F_ST,_ calculated using *vcftools*^64^, and CNV tracks. The 90% truth VSQR-filtered ‘PASS’ data were used for all *vcftools* calculations, allowing only bi-allelic SNPs and a maximum of 20% of missing data per SNP. The output tables were converted to bigWig format using the *BigWig* utilities^65^. For the CNV tracks we used *bedtools*^66^ to intersect calls from all individuals belonging to the same population. Within each population average copy number across individuals was calculated for every given interval and transformed to log_2_ values. The genomic browser tracks provided at the ftp site allow a visualization of differences between the populations and species for genomic regions of interest.

For RNAseq data, the coverage (number of reads at a given base pair) is expected to be proportional to the expression level of the gene from which the read originated. In order to compare RNAseq coverage (and thus gene expression) across individuals with slightly varying total numbers of mapped reads, we normalized the data by proportionally sub-sampling reads from the alignment file (*.bam) for the individuals with higher numbers of total mapped reads. Specifically, for each given tissue, we first determined the individual with the lowest number of total mapped reads. This individual is assigned the normalization factor of 1. For each additional individual we calculated the factor c, total number of mapped reads in the individual with the lowest number / total number of reads in sampled individual. Values of c range from 0 to <1. Thus, the individual with the highest number of mapped reads will have a value of c closest to zero. This normalization factor is then used with the *samtools view –s x.y* command, where x=0 and y=the decimal part of the normalization factor c to generate the normalized alignment file.

**Figure 3:**
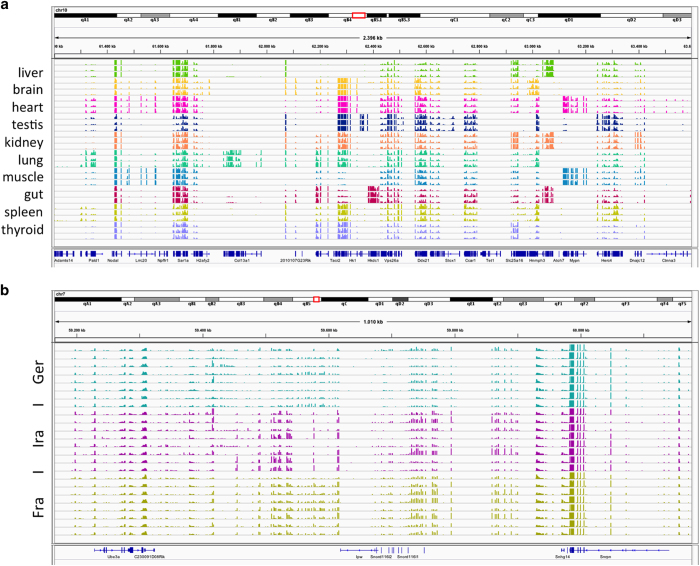
Examples of IGV browser views for the transcriptome data. (**a**) Region chr10:61,200,000–63,600,000 in the mouse *mm10* genome displaying results for all tissues with the data combined from all individuals of the three *M. m. domesticus* populations. The population order is for each tissue Germany-Iran-France from top. (**b**) Region chr7:59,165,000–60,177,000 in the *mm10* mouse genome, displaying results for brain normalized RNAseq read coverage with all individuals displayed for each of the three *M. m. domesticus* populations. Note that there is RNAseq read coverage (i.e., ‘gene expression’) between the annotated coding genes, a region corresponding to known non-coding snoRNAs located within tandem repeats. This expression is limited to the brain among the tissues sampled. Expression differences between the populations are evident.

To visualize the data as number of reads covering a particular location (i.e., base pair) in the genome, we converted normalized alignment files into bedgraph files, which were further compressed into bigWig format (available on the ftp site). The RNAseq based read coverage for each basepair in the genome can then be visualized in the UCSC (available as public session under ‘wildmouse’) or IGV browsers^46,47^. Figure 3 shows two examples of screen shots from IGV sessions. The first is a general overview across all tissues from a section of chromosome 10. The second shows only a single tissue (brain), but with read coverage information for each individual.

## Data Records

The primary read files for the genome sequences are available at the European Nucleotide Archive (ENA) under project accession number PRJEB9450 (Data Citation 1) for the *M. m. domesticus* genomes, under project accession number PRJEB11742 (Data Citation 2) and PRJEB14167 (Data Citation 3) for the *M. m. musculus* and the *M. spretus* genomes and under project accession number PRJEB2176 (Data Citation 4) for the *M. m. castaneus* genomes processed in this study. All genome samples and their associated sample designations are listed in Table 1 (available online only). The transcriptome read files are available at ENA under project accession number PRJEB11897 (Data Citation 5). The samples and their sample designation are described in Table 2 (available online only).

The files with the mapped reads (bam), variant calling (vcf) and browser tracks (bigWig) for the genomes and transcriptomes, as well as the IGV session files for the transcriptomes are available at:

http://wwwuser.gwdg.de/~evolbio/evolgen/wildmouse/ where they can be accessed via ftp.

## Technical Validation

We used the software *angsd*^67^ and its -doDepth 1 command (with options -minMapQ 30 -minQ 20) to assess the quality of each DNA sequencing library with respect to good quality coverage (both mapping and base quality) of the genome. The *angsd* depth analysis is based on all sequenced and mapped bases, rather than only on called genotypes at variable sites. Thus, this is the most comprehensive way to assess coverage across the whole genome. The average per-base coverage for each sequenced genome is given in Table 1 (available online only) for autosomes, the X-chromosome and the Y chromosome separately. It was calculated as: $\frac{\sum_{\mathrm{i=1}}^{60}n_{i}\times i}{\text{genome size}}$, where n_i_ is number of bases sequenced at depth *i* and genome size is 2,395,908,738 for autosomes, 163,487,995 for the X chromosome and 88,124,698 for the Y chromosome.

The autosomal coverage is variable across individuals (both within and between sequencing batch) but should be high enough to obtain good quality SNP calls. For males, the X-chromosome coverage is expected to be half of the autosomal coverage, while for females, the coverage should be similar for X chromosome and autosomes. We can use this fact to obtain independent confirmation of the animal’s sex recorded in the field. For all but one case, the genomic sex (based on X/autosome coverage ratio) matched the sex determined in the field. Individual AL42 was recorded as male in the field, but its genomic data clearly suggest it was a female. For juvenile wild mice it can sometimes be difficult to accurately determine their sex. The lower X-chromosomal coverage for males indicates that some caution should be taken when using called genotypes for this chromosome for population genetic inferences. Approaches that take the genotype likelihoods into account to estimate parameters may be better suited for the X-chromosomal data (i.e., ref. 67). The Y chromosome is extremely poorly covered (see below).

We also assessed the uniformity of coverage across the genome, aiming at identifying specific regions, where few or no reads could be mapped. We ran the *angsd* -doDepth 1 command for non-overlapping 100 kb windows recording the average (across individuals within subspecies) % bases covered at >10 reads/individual in 100 kb windows. As shown in Supplementary Fig. 4 using all *M. m. domesticus* individuals as an example, there are some regions in the genome where coverage is low or absent. This pattern was highly correlated in the other subspecies/species (data not shown), suggesting that features of the reference genome (possibly presence of repetitive elements or un-sequenced parts of the reference genome) limit mapping of reads in these regions. Regional variation in coverage is especially striking on the Y-chromosome, where coverage is limited to several short regions within the proximal 10 Mb of the chromosome. This region roughly corresponds to the male-specific short arm of the Y chromosome and its centromere.

### Confirmation that mice are naturally inbred

The natural inbreeding status of wild caught mice is expected to be influenced by population history, as well as their tendency to form extended family structures with breeding among relatives^48,49^. The degree of inbreeding is expected to be higher for small populations and also for populations that recently colonized new habitats, a process which often involves a bottleneck. For the *M. musculus* mice included in this dataset, Iranian, Afghanistan and Indian mice are closest to the center of origin of this species and thus are expected to be least inbred, as such populations are expected to have been large and stable over time. On the other hand, *M. m. helgolandicus* inhabits a very small island and experienced a strong founder event during colonization^26^. For *Mus spretus*, we do not have a good expectation, as wild populations of this species have never been studied. It is generally assumed that *M. spretus* does not form extended family structures, as these mice are not human commensals but live in fields, hedges and boundaries to forests.

**Figure 4:**
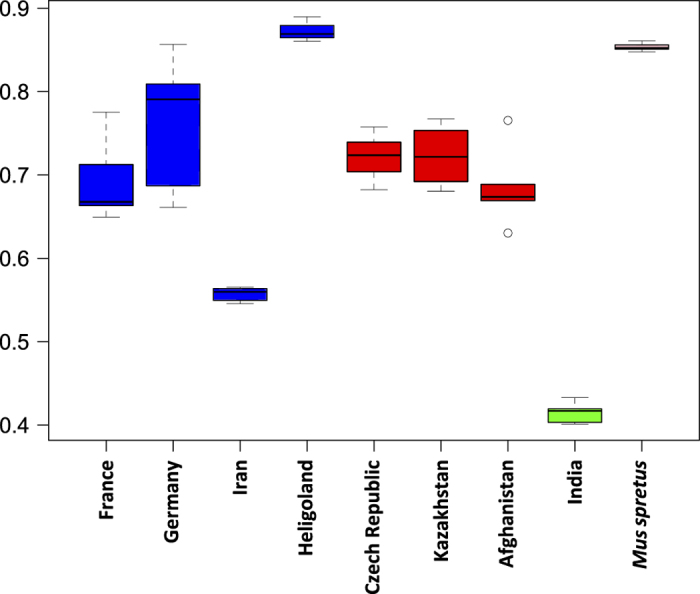
Distribution of inbreeding coefficients within populations. *M. m. domesticus* and *M. m. helgolandicu*s populations are highlighted in blue, *M. m. musculus* populations are highlighted in red, the *M. m. castaneus* population is highlighted in green and *Mus spretus* is highlighted in purple.

We used a combination of *angsd*^67^ to calculate genotype likelihoods and *ngsF*^68^ to calculate inbreeding coefficients for each individual based on randomly selected 1,000 autosomal 10 kb fragments (see Supplementary Material for scripts). As shown in Fig. 4, our expectations are mostly met, with ancestral populations from India and Iran showing the lowest estimated inbreeding coefficient, while Heligoland individuals are close to representing inbred lines. Surprisingly, the *Mus spretus* individuals from Spain are highly inbred, suggesting that they may have experienced a recent bottleneck.

### Confirmation that animals cluster with their respective population

**Figure 5:**
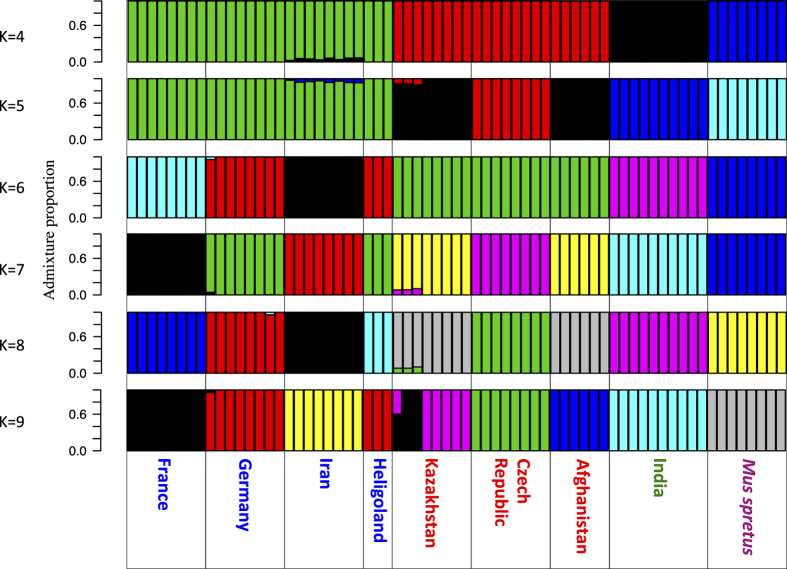
Estimated cluster membership and admixture proportions. Plots for each individual in the sequencing study, for K=4 to K=9 (number of assumed populations). Individuals are sorted by population and subspecies. *M. m. domesticus* populations are highlighted in blue, *M. m. musculus* populations are highlighted in red, the *M. m. castaneus* population is highlighted in green and *Mus spretus* is highlighted in purple.

The aim of this analysis was to confirm that each sequenced individual is assigned to its respective population, based on its SNP genotypes. We used the software *NgsAdmix*^69^ on the same randomly selected 1,000 autosomal 10 kb fragments as used in the previous analysis. As before, genotype likelihoods were generated by *angsd*. We ran the *NgsAdmix* software for K=1 to K=9 (Fig. 5). The likelihood of the data increases dramatically from K=1 to K=4 and then plateaus (data not shown). Such a pattern (see ref. 70) is usually taken as evidence that K=4 fits the data best. K=4 clusters all individuals with their correct subspecies (*M. m. helgolandicus* correctly clusters with *M. m. domesticus*^26^) and species respectively. At K=7, we can subdivide the *M. m. domesticus* subspecies into its respective populations (with *M. m. helgolandicus* clustering with the German individuals, confirming the previous results based on microsatellites^26^). For the *M. m. musculus* subspecies we find the individuals from the Czech Republic to split off from those from Afghanistan and Kazakhstan. The latter two populations seem to be more closely related. Generally, the genetic clustering analysis suggests that the populations are well defined and differentiated and that there are no recent immigrants from other areas among the sequenced individuals (note that the seeming admixture in K=9 is an artifact of too high K).

### Confirmation that VSQR 90 tranche filtering yields expected levels of polymorphism

We assessed the GATK VSQR 90% tranche PASS-filtered SNPs for levels of polymorphism (Watterson’s θ^71^) within each population and compared the estimates to several reference data sets, which have previously been generated using Sanger sequencing on smaller number of loci. The Indian population is especially informative, as it has been extensively sequenced^51,72^. For each chromosome and each population we determined the number of segregating sites using the software *PopGenome*^73^. Values were summarized over the autosomal genome and converted into Watterson’s θ in % by dividing by a_i_^71^ and the number of sites sequenced (Supplementary Table 4). The resulting θ_W_/bp in % for the Indian population (0.74) lies in between the value obtained by ref. 51 (0.91, for 4-fold degenerated sites and 0.83 for intronic sites) and the one obtained by ref. 72 for the same Indian population (0.664). θ_W_ for the Western European *M. m. domesticus* individuals was 0.213 in ref. 72, 0.18 for our German population and 0.2 for our French population. Thus, overall, the VSQR 90% PASS-filtered SNPs dataset seem to reflect the previously inferred levels of polymorphism of house mouse populations quite well.

### Analysis of relatedness in the sample

We used the —relatedness2 option of *vcftools* to assess pairwise individual relatedness among all mice in the dataset, using the KING method^74^. This analysis is based on GATK called genotypes and the 90% tranche PASS-filtered SNPs. We restricted the dataset to only include autosomal SNPs, thinned to 1 SNP every 1 Mb. We also removed sites that had more than 20% missing data and only included bi-allelic markers in the analysis. As described in Table 1 of ref. 74, expected ranges of kinship coefficients (‘Phi’) are >0.354 for duplicate samples/monozygotic twins, [0.177–0.354] for 1st degree relatives, [0.0884–0.177] for 2nd degree relative, [0.0442–0.0884] for 3rd degree relatives and <0.0442 for unrelated samples. Out of 2,211 pairwise individual relatedness estimates, 35 indicated first (10 pairwise comparisons), second (three pairwise comparisons) and third degree (22 pairwise comparisons) relatives (Supplementary Table 5). However, since we detected third degree relationship also among animals that were unequivocally caught far apart (e.g., SP39-SP68), we only consider first and second degree relatedness relevant here. No duplicate samples were detected (expected Phi=0.5). Relatedness was only detected within populations, and was absent between them. No first or second-degree relatedness was found for the German *M. m. domesticus*, the Afghanistan *M. m. musculus* and the Indian *M. m. castaneus* populations. Most related animals were found in the populations from Iran and Kazakhstan. In the case of the Iranian population the increased relatedness within the sample can be explained by the fact that some breeding adults were used in multiple crosses (see Supplementary Table 2). The relatedness observed in the population from Kazakhstan is best explained by the fact that mice were collected in close proximity, rather than over a larger regional scale.

We can use the known breeding setup of the Iranian mice to confirm the inferred relatedness categories in this population. The KING method detected 2 first-degree relationships in the Iranian population. Male AH15 is indeed the father of JR5-F1C. However, JR-7F1C is the brother of the mother (i.e., uncle) of JR11 and thus a second-degree relative. The second-degree relative identified by the KING method in the Iranian sample is consistent with the breeding scheme, with JR11 and JR15 being half siblings (they have the same father). Two third degree relationships are incorrectly identified and should be second-degree relationships instead (i.e., JR5-F1C is the uncle of JR15 and AH15 is grandfather of JR15) and one third-degree relationship does not have any known breeding history confirming it. The KING method assumes Hardy-Weinberg equilibrium among SNPs with the same underlying allele frequencies. This assumption is most likely being violated in our dataset (given that wild mice are generally inbred, see above), and could explain some of the miss-assignments between categories.

### Confirmation of known t-haplotype carriers and identifying t-haplotype carriers in the total dataset

The t-haplotype is complex set of 4 inversions, comprising a 20 cM (30–40 Mbp) region of the proximal third of chromosome 17 in house mice^75^. It is a selfish genetic element, which causes transmission ratio distortion, with heterozygous t-haplotype carriers predominantly (sometimes up to 99% of times) transmitting the t-haplotype carrying chromosome to their offspring. Homozygous individuals for the t-haplotype, however, die *in utero*. Despite their massive transmission advantage, t-haplotype carrying individuals are rare in natural populations of mice, but have been found in all recognized subspecies.

We have previously used two published primer pairs^76^ to genotype individuals in our collections of mouse samples for presence/absence of the t-haplotype. Both primer pairs span t-haplotype diagnostic indels that can be analyzed on an agarose gel (the proximal locus *Tcp1* spans a 175 bp indel and the distal locus *Hba-4ps* spans a 16 bp indel). Three individuals typed with those primers are identical to samples included in the whole genome sequencing study described here: male AL41, male CR16 and female H14. Of those, AL41 and H14 were found to be carriers (heterozygous) of the t-haplotype, while CR16 was wildtype. We use ENSEMBL Blast to determine the location of those primers in the *mm10* reference sequence. All primers yielded unique hits. We then extracted all indels spanning the region between the primer locations for all samples from the .vcf-file, and searched for (combinations of) indels matching the sizes above. For the distal locus, we found a single 17 bp indel at position chr17:26,286,509 (‘ TACTACTATGCACTGAA’). For the proximal locus, we found one indel at position chr17:12,921,682 that was identified as ‘ GTTTTTTTTTTT’. Illumina sequencing is not capable to sequence through long homo-polymer stretches which is likely the reason why the identified indel is reported shorter than the expected 175 bp. However, it is the only indel >3 bp in the region and moreover, genotypes at this indel are in almost perfect linkage disequilibrium with genotypes at the distal locus (see Table 1 (available online only)), which is highly unexpected over a distance of 13.3 Mb. The two individuals that we previously found to be positive for the t-haplotype using the PCR primers are also heterozygous for the respective indels in the whole genome dataset, while CR16 was wildtype based on the whole genome sequence. Thus, indels at chr17:12,921,682 and chr17:26,286,509 were used to genotype the remaining individuals in the dataset for the presence/absence of t-haplotypes. All *M. musculus* individuals positive for the *t*-haplotype indels were heterozygous for that *t*-allele. *Mus spretus*, on the other hand, was homozygous for the proximal t-specific indel, which is consistent with a rather old origin of the t-allele (1–3 Myr^77^), despite not having any obvious transmission ratio distortion properties in a species outside the *M. musculus* complex. *t*^*+*^/*wt* individuals were found in every population apart from the Iranian population and among the three individuals from Heligoland. T-haplotype carriers reached frequencies of 50% in two *M. musculus* populations (Afghanistan and Czech Republic). 37,5% of the French individuals carried the t-haplotype, as did 30% of Indian individuals, 25% of individuals from Kazakhstan and 12.5% of the German population. The frequency of t-haplotypes in our data is somewhat higher than reported previously (reviewed in ref. 78), however well below the theoretical expectations based on transmission ratio distortion (see ref. 79).

### K-mer distributions to determine complexity of RNAseq libraries

We analyzed the *k*-mer (DNA sequence stretch of length *k*) frequency spectrum to identify potential problems with the RNAseq libraries, such as DNA contamination and overabundance (potentially due to PCR amplification) of particular sequence stretches. Ideally, libraries are complex, meaning they exhibit great diversity in unique sequences and repeated structures^80^ and there should be no sign of DNA contamination among the RNA based reads generated. In total, we generated 224 RNAseq libraries from up to 10 tissues in 24 *M. m. domesticus* individuals. For each library, we ran the software *jellyfish*^81^ on all forward reads with a k-mer length of k=12. Using reverse reads yielded the same results (data not shown). For visualization in Supplementary Fig. 5 we randomly choose three individuals from each population and three tissues (brain, testis and liver). The complete set of all 224 RNAseq k-mer distributions are available on our ftp server. The k-mer distribution of the mouse *mm10* DNA sequence (red in Supplementary Fig. 5) produces a characteristic line with 2 prominent humps when plotted on a log10–log10 scale. The k-mer distribution for annotated cDNAs in the mouse genome (ENSEMBL version 83 (ref. 61)) does not produce such humps and compared to the DNA *mm10* sequence is more ‘complex’, i.e., shows proportionally more unique sequences (with low 12-mer occurrence, but high frequency, left side of plots in Supplementary Fig. 5). The k-mer distribution of the RNAseq forward reads (grey in Supplementary Fig. 5) falls in between the cDNA and genomic DNA profile. Since we generated our RNAseq libraries from poly-adenylated RNAs, comparing the RNAseq profile to annotated cDNA seems appropriate. Most notably, the RNAseq k-mer profile lacks the characteristic humps of the genomic DNA k-mer profile, suggesting that the RNA libraries are not contaminated with DNA. Moreover, the diversity of unique sequences matched the cDNA profile much better than the DNA profile. Very similar patterns have been observed for human RNAseq data, that have been deemed good quality (see Supplementary Fig. 6A in ref. 80).

## Additional information

**How to cite this article:** Harr, B. *et al.* Genomic resources for wild populations of the house mouse, *Mus musculus* and its close relative *Mus spretus*. *Sci. Data* 3:160075 doi: 10.1038/sdata.2016.75 (2016).

## Supplementary Material



Supplementary Table 1

Supplementary Table 2

Supplementary Table 3

Supplementary Table 4

Supplementary Table 5

Supplementary Material

## Figures and Tables

**Table 1:** Overview of DNA sequencing data

**Species**	**Population - map location**	**Population ID**	**Population ID**	**Field sex**	**Sample ID**	**sequencing batch**	**total reads (x108)**	**high quality mapped (%)**	**fold coverage Autosomes**	**fold coverage X chromosome**	**fold coverage Y chromosome**	**X/A ratio** *	**t-haplotype locus** ***Tcp1*** **chr17:12,921,682**	**t-haplotype locus*****Hba-4ps*** **chr17:26,286,509**	**ENA study accession number**	**ENA sample accession number**
Mus musculus domesticus	France (Massif Central) - 1	MC	FRA1	male	14	1	7.35	95.5	24	12	0.60	0.5	0/0	0/0	PRJEB9450	ERS739386
Mus musculus domesticus	France (Massif Central) - 1	MC	FRA2	male	15B	1	7.18	94.5	23	11	0.59	0.5	0/0	0/0	PRJEB9450	ERS739387
Mus musculus domesticus	France (Massif Central) - 1	MC	FRA3	male	16B	1	7.46	94.7	24	12	0.61	0.5	0/0	0/0	PRJEB9450	ERS739388
Mus musculus domesticus	France (Massif Central) - 1	MC	FRA4	male	18B	1	7.3	96.1	24	11	0.61	0.5	0/0	0/0	PRJEB9450	ERS739381
Mus musculus domesticus	France (Massif Central) - 1	MC	FRA5	male	B2C	1	4.75	91.9	14	7	0.44	0.5	0/1	0/1	PRJEB9450	ERS739383
Mus musculus domesticus	France (Massif Central) - 1	MC	FRA6	male	C1	1	6.18	94.3	20	9	0.53	0.5	0/1†	0/0	PRJEB9450	ERS739384
Mus musculus domesticus	France (Massif Central) - 1	MC	FRA7	male	E1	1	6.72	94.9	22	10	0.54	0.5	0/0	0/0	PRJEB9450	ERS739385
Mus musculus domesticus	France (Massif Central) - 1	MC	FRA8	male	F1B	1	6.93	95.7	23	11	0.58	0.5	0/1	0/1	PRJEB9450	ERS739382
Mus musculus domesticus	Germany (Cologne-Bonn) - 2	CB	GER1	male	TP1	1	6.84	94.5	23	11	0.55	0.5	0/0	0/0	PRJEB9450	ERS739373
Mus musculus domesticus	Germany (Cologne-Bonn) - 2	CB	GER2	male	TP121B	1	6.56	95.6	22	10	0.54	0.5	0/0	0/0	PRJEB9450	ERS739379
Mus musculus domesticus	Germany (Cologne-Bonn) - 2	CB	GER3	male	TP17-2	1	7.01	96	24	11	0.57	0.5	0/0	0/0	PRJEB9450	ERS739376
Mus musculus domesticus	Germany (Cologne-Bonn) - 2	CB	GER4	male	TP3-92	1	7.04	95.1	23	11	0.58	0.5	0/0	0/0	PRJEB9450	ERS739374
Mus musculus domesticus	Germany (Cologne-Bonn) - 2	CB	GER5	male	TP4a	1	7.21	95.5	24	11	0.58	0.5	0/0	0/0	PRJEB9450	ERS739375
Mus musculus domesticus	Germany (Cologne-Bonn) - 2	CB	GER6	male	TP51D	1	6.25	94.4	20	10	0.53	0.5	0/1	0/1	PRJEB9450	ERS739377
Mus musculus domesticus	Germany (Cologne-Bonn) - 2	CB	GER7	male	TP7-10F1A2	1	6	94.8	20	9	0.52	0.5	0/0	0/0	PRJEB9450	ERS739380
Mus musculus domesticus	Germany (Cologne-Bonn) - 2	CB	GER8	male	TP81B	1	6.61	94.8	22	10	0.54	0.5	0/0	0/0	PRJEB9450	ERS739378
Mus musculus domesticus	Germany (Heligoland) - 3	HEL	HEL1	female	HG06	2	3.26	96	11	10	0.01	0.9	0/0	0/0	PRJEB9450	ERS739725
Mus musculus domesticus	Germany (Heligoland) - 3	HEL	HEL2	male	HG08	2	4.09	96.4	14	6	0.41	0.5	0/0	0/0	PRJEB9450	ERS739726
Mus musculus domesticus	Germany (Heligoland) - 3	HEL	HEL3	female	HG13	2	3.57	96.7	12	10	0.01	0.9	0/0	0/0	PRJEB9450	ERS739727
Mus musculus domesticus	Iran (Ahvaz) - 4	AH	IRA1	male	AH15	1	7.02	91.6	22	10	0.59	0.5	0/0	0/0	PRJEB9450	ERS739396
Mus musculus domesticus	Iran (Ahvaz) - 4	AH	IRA2	male	AH23	1	8.07	89.6	24	11	0.58	0.4	0/0	0/0	PRJEB9450	ERS739395
Mus musculus domesticus	Iran (Ahvaz) - 4	AH	IRA3	male	JR11	1	7.66	91.2	23	11	0.59	0.5	0/0	0/0	PRJEB9450	ERS739393
Mus musculus domesticus	Iran (Ahvaz) - 4	AH	IRA4	male	JR15	1	7	93.1	22	11	0.57	0.5	0/0	0/0	PRJEB9450	ERS739394
Mus musculus domesticus	Iran (Ahvaz) - 4	AH	IRA5	male	JR2-F1C	1	7	95	23	11	0.57	0.5	0/0	0/0	PRJEB9450	ERS739389
Mus musculus domesticus	Iran (Ahvaz) - 4	AH	IRA6	male	JR5-F1C	1	5.79	90.6	17	8	0.55	0.5	0/0	0/0	PRJEB9450	ERS739390
Mus musculus domesticus	Iran (Ahvaz) - 4	AH	IRA7	male	JR7-F1C	1	6.06	92.2	18	8	0.52	0.5	0/0	0/0	PRJEB9450	ERS739391
Mus musculus domesticus	Iran (Ahvaz) - 4	AH	IRA8	male	JR8-F1A	1	5.5	94.2	17	8	0.47	0.5	0/0	0/0	PRJEB9450	ERS739392
Mus musculus musculus	Czech Republic (Studenec) - 5	CR	CZE1	female	CR12	3	8.12	92.4	25	21	0.02	0.9	0/1	0/1	PRJEB11742	ERS957496
Mus musculus musculus	Czech Republic (Studenec) - 5	CR	CZE2	female	CR13	3	7.74	92.7	24	20	0.02	0.9	0/1	0/1	PRJEB11742	ERS957497
Mus musculus musculus	Czech Republic (Studenec) - 5	CR	CZE3	male	CR16	3	8.1	92.6	25	11	0.76	0.4	0/0	0/0	PRJEB11742	ERS957498
Mus musculus musculus	Czech Republic (Studenec) - 5	CR	CZE4	female	CR23	3	7.95	92.9	24	20	0.02	0.8	0/1	0/1	PRJEB11742	ERS957499
Mus musculus musculus	Czech Republic (Studenec) - 5	CR	CZE5	female	CR25	3	8.08	92.4	25	20	0.02	0.8	0/0	0/0	PRJEB11742	ERS957500
Mus musculus musculus	Czech Republic (Studenec) - 5	CR	CZE6	female	CR29	3	7.37	92.7	23	19	0.02	0.8	0/0	0/1†	PRJEB11742	ERS957501
Mus musculus musculus	Czech Republic (Studenec) - 5	CR	CZE7	male	CR46	3	7.98	92.2	24	11	0.76	0.4	0/0	0/0	PRJEB11742	ERS957502
Mus musculus musculus	Czech Republic (Studenec) - 5	CR	CZE8	female	CR59	3	8.16	92.7	25	21	0.02	0.8	0/0	0/0	PRJEB11742	ERS957503
Mus musculus musculus	Kazhakstan (Almaty) - 6	KAZ	KAZ1	female	AL1	3	7.66	92.4	23	20	0.02	0.9	0/0	0/0	PRJEB11742	ERS957504
Mus musculus musculus	Kazhakstan (Almaty) - 6	KAZ	KAZ2	male	AL16	3	8.23	92.8	25	11	0.02	0.4	0/0	0/0	PRJEB11742	ERS957505
Mus musculus musculus	Kazhakstan (Almaty) - 6	KAZ	KAZ3	female	AL19	3	7.98	92.8	24	20	0.97	0.8	0/1	0/1	PRJEB11742	ERS957506
Mus musculus musculus	Kazhakstan (Almaty) - 6	KAZ	KAZ4	female	AL33	3	8.16	93	25	21	0.02	0.8	0/0	0/0	PRJEB11742	ERS957507
Mus musculus musculus	Kazhakstan (Almaty) - 6	KAZ	KAZ5	male	AL38	3	8.04	92.8	25	11	0.95	0.4	0/0	0/0	PRJEB11742	ERS957508
Mus musculus musculus	Kazhakstan (Almaty) - 6	KAZ	KAZ6	female	AL40	3	8.54	92.8	26	22	0.02	0.9	0/0	0/0	PRJEB11742	ERS957509
Mus musculus musculus	Kazhakstan (Almaty) - 6	KAZ	KAZ7	male	AL41	3	8.37	92.8	26	11	1.00	0.4	0/1	0/1	PRJEB11742	ERS957510
Mus musculus musculus	Kazhakstan (Almaty) - 6	KAZ	KAZ8	male	AL42	3	8.26	92.6	25	22	0.02	0.9	0/0	0/0	PRJEB11742	ERS957511
Mus musculus musculus	Afghanistan (Mazar-e-Sharif) - 7	AFG	AFG1	male	396	4	6.68	97.08	14	6	0.43	0.4	0/1	0/1	PRJEB14167	ERS1180808
Mus musculus musculus	Afghanistan (Mazar-e-Sharif) - 7	AFG	AFG2	male	413	4	11.96	96.05	21	9	0.69	0.4	0/0	0/0	PRJEB14167	ERS1180809
Mus musculus musculus	Afghanistan (Mazar-e-Sharif) - 7	AFG	AFG3	male	416	4	9.46	96.01	16	6	0.48	0.4	0/1	0/1	PRJEB14167	ERS1180810
Mus musculus musculus	Afghanistan (Mazar-e-Sharif) - 7	AFG	AFG4	male	424	4	10.12	95.12	17	7	0.56	0.4	0/0	0/0	PRJEB14167	ERS1180811
Mus musculus musculus	Afghanistan (Mazar-e-Sharif) - 7	AFG	AFG5	female	435	4	12.17	96.65	19	16	0.03	0.8	0/0	0/0	PRJEB14167	ERS1180812
Mus musculus musculus	Afghanistan (Mazar-e-Sharif) - 7	AFG	AFG6	male	444	4	11.46	94.62	18	7	0.58	0.4	0/1	0/1	PRJEB14167	ERS1180813
Mus musculus castaneus	India (Himalaya) - 8	CAS	CAST1	male	H12	na	6.12	91.2	20	9	0.53	0.4	0/1	0/1	PRJEB2176	ERS003051
Mus musculus castaneus	India (Himalaya) - 8	CAS	CAST2	female	H14	na	5.66	88.5	17	16	0.01	0.9	1/2	0/1	PRJEB2176	ERS003041
Mus musculus castaneus	India (Himalaya) - 8	CAS	CAST3	female	H15	na	4.1	89.6	13	11	0.01	0.8	0/0	0/0	PRJEB2176	ERS003045
Mus musculus castaneus	India (Himalaya) - 8	CAS	CAST4	female	H24	na	4.52	84.9	13	12	0.01	0.9	0/0	0/0	PRJEB2176	ERS003042
Mus musculus castaneus	India (Himalaya) - 8	CAS	CAST5	female	H26	na	5.67	87.7	17	15	0.01	0.9	0/0	0/0	PRJEB2176	ERS003046
Mus musculus castaneus	India (Himalaya) - 8	CAS	CAST6	female	H27	na	4.47	88.8	13	12	0.01	0.9	0/1	0/1	PRJEB2176	ERS003047
Mus musculus castaneus	India (Himalaya) - 8	CAS	CAST7	male	H28	na	4.82	90.6	15	7	0.45	0.5	0/0	0/0	PRJEB2176	ERS003048
Mus musculus castaneus	India (Himalaya) - 8	CAS	CAST8	female	H30	na	7.03	87.3	21	17	0.02	0.8	0/0	0/0	PRJEB2176	ERS003044
Mus musculus castaneus	India (Himalaya) - 8	CAS	CAST9	male	H34	na	6.63	91.7	21	10	0.57	0.5	0/0	0/0	PRJEB2176	ERS003049
Mus musculus castaneus	India (Himalaya) - 8	CAS	CAST10	female	H36	na	6.38	90	19	16	0.03	0.8	0/0	0/0	PRJEB2176	ERS003050
Mus spretus	Spain (Madrid) - S	SPRE	SPRE1	male	SP36	3	7.74	89.4	21	9	0.24	0.4	1/1	0/0	PRJEB11742	ERS957512
Mus spretus	Spain (Madrid) - S	SPRE	SPRE2	male	SP39	3	8.7	91.2	23	10	0.27	0.4	1/1	0/0	PRJEB11742	ERS957513
Mus spretus	Spain (Madrid) - S	SPRE	SPRE3	male	SP41	3	8.14	91.4	22	9	0.26	0.4	1/1	0/0	PRJEB11742	ERS957514
Mus spretus	Spain (Madrid) - S	SPRE	SPRE4	female	SP51	3	8.25	91.3	22	17	0.02	0.8	1/1	0/0	PRJEB11742	ERS957515
Mus spretus	Spain (Madrid) - S	SPRE	SPRE5	female	SP62	3	8.27	91.3	23	18	0.02	0.8	1/1	0/0	PRJEB11742	ERS957516
Mus spretus	Spain (Madrid) - S	SPRE	SPRE6	male	SP68	3	8.06	91.5	22	9	0.26	0.4	1/1	0/0	PRJEB11742	ERS957517
Mus spretus	Spain (Madrid) - S	SPRE	SPRE7	male	SP69	3	8.34	90.9	22	9	0.29	0.4	1/1	0/0	PRJEB11742	ERS957518
Mus spretus	Spain (Madrid) - S	SPRE	SPRE8	male	SP70	3	7.94	91.2	22	9	0.27	0.4	1/1	0/0	PRJEB11742	ERS957519

*0.4–0.5: male 0.8–0.9: female

^†^partial t-haplotype

**Table 2:** Summary of transcriptome data for 8 tissues in three *M. m. domesticus* populations

**Population - map location**	**Population ID**	**Population ID**	**sex**	**Genome Sample ID**	**transcriptome Sample ID**	**available tissues**	**Mapped reads Brain**	**proportion of mapped reads Brain (%)**	**ENA sample accession**	**Mapped reads Gut**	**proportion of mapped reads Gut (%)**	**ENA sample accession**	**Mapped reads Heart**	**proportion of mapped reads Heart (%)**	**ENA sample accession**	**Mapped reads Kidney**	**proportion of mapped reads Kidney (%)**	**ENA sample accession**	**Mapped reads Liver**	**proportion of mapped reads Liver (%)**	**ENA sample accession**	**Mapped reads Lung**	**proportion of mapped reads Lung (%)**	**ENA sample accession**	**Mapped reads Muscle**	**proportion of mapped reads (%) Muscle**	**ENA sample accession**	**Mapped reads Testis**	**proportion of mapped reads Testis (%)**	**ENA sample accession**	**Mapped reads Spleen**	**proportion of mapped reads Spleen (%)**	**ENA sample accession**	**Mapped reads Thyroid**	**proportion of mapped reads Tyroid (%)**	**ENA sample accession**
France (Massif Central) - 1	MC	FRA1	male	14	14	9	2.75E+07	(95.0%)	ERS986056	NA	NA	NA	2.56E+07	(95.8%)	ERS986071	3.22E+07	(95.4%)	ERS986080	4.26E+07	(89.7%)	ERS986088	3.01E+07	(95.0%)	ERS986097	3.43E+07	(95.6%)	ERS986101	3.62E+07	(94.7%)	ERS986118	3.08E+07	(94.1%)	ERS986110	2.49E+07	(91.1%)	ERS986059
France (Massif Central) - 1	MC	FRA2	male	15B	15b	10	2.40E+07	(95.5%)	ERS986057	2.60E+07	(95.0%)	ERS986065	3.47E+07	(96.0%)	ERS986072	3.13E+07	(95.1%)	ERS986081	3.24E+07	(91.8%)	ERS986090	NA	NA	NA	2.97E+07	(96.1%)	ERS986102	3.25E+07	(90.9%)	ERS985990	3.42E+07	(95.0%)	ERS986111	2.30E+07	(92.1%)	ERS986044
France (Massif Central) - 1	MC	FRA3	male	16B	16b	9	3.20E+07	(95.5%)	ERS986058	2.43E+07	(94.3%)	ERS986066	3.11E+07	(96.1%)	ERS986073	3.01E+07	(95.1%)	ERS986082	3.77E+07	(91.4%)	ERS986091	NA	NA	NA	3.70E+07	(95.4%)	ERS986103	3.77E+07	(91.1%)	ERS985991	3.55E+07	(95.2%)	ERS986112	2.47E+07	(90.9%)	ERS986029
France (Massif Central) - 1	MC	FRA4	male	18B	18b	9	3.31E+07	(95.6%)	ERS986060	2.87E+07	(93.7%)	ERS986067	3.22E+07	(96.1%)	ERS986075	3.28E+07	(95.7%)	ERS986083	4.08E+07	(89.4%)	ERS986092	3.27E+07	(94.7%)	ERS986098	3.44E+07	(95.8%)	ERS986105	3.42E+07	(91.3%)	ERS986005	3.31E+07	(94.6%)	ERS986113	2.21E+07	(89.9%)	ERS985984
France (Massif Central) - 1	MC	FRA5	male	B2C	B2C	9	2.98E+07	(95.5%)	ERS986061	2.91E+07	(94.4%)	ERS986068	3.09E+07	(95.7%)	ERS986076	3.49E+07	(95.3%)	ERS986084	3.78E+07	(90.2%)	ERS986093	NA	NA	NA	3.33E+07	(95.6%)	ERS986106	3.72E+07	(95.1%)	ERS986119	3.47E+07	(95.3%)	ERS986114	2.11E+07	(89.7%)	ERS985969
France (Massif Central) - 1	MC	FRA6	male	C1	C12	10	3.17E+07	(95.7%)	ERS986062	2.48E+07	(94.2%)	ERS986069	3.05E+07	(95.8%)	ERS986077	2.90E+07	(95.7%)	ERS986085	3.84E+07	(90.5%)	ERS986094	3.11E+07	(94.8%)	ERS986099	3.35E+07	(95.3%)	ERS986107	3.56E+07	(94.8%)	ERS986104	3.16E+07	(95.6%)	ERS986115	2.36E+07	(90.4%)	ERS985954
France (Massif Central) - 1	MC	FRA7	male	E1	E1b	10	3.02E+07	(95.8%)	ERS986063	3.08E+07	(95.0%)	ERS986070	3.09E+07	(95.8%)	ERS986078	3.75E+07	(95.2%)	ERS986086	3.83E+07	(91.8%)	ERS986095	3.30E+07	(95.3%)	ERS986100	3.53E+07	(95.4%)	ERS986108	3.58E+07	(94.9%)	ERS986089	3.11E+07	(95.1%)	ERS986116	2.50E+07	(90.1%)	ERS985939
France (Massif Central) - 1	MC	FRA8	male	F1B	F1B	8	3.08E+07	(95.6%)	ERS986064	NA	NA	NA	3.73E+07	(95.8%)	ERS986079	3.37E+07	(95.4%)	ERS986087	3.37E+07	(91.9%)	ERS986096	NA	NA	NA	3.42E+07	(95.7%)	ERS986109	3.78E+07	(94.9%)	ERS986074	3.16E+07	(94.8%)	ERS986117	3.43E+07	(87.5%)	ERS986014
Germany (Cologne-Bonn) - 2	CB	GER1	male	TP1	tp1	10	2.44E+07	(95.2%)	ERS985899	2.51E+07	(92.3%)	ERS985907	3.21E+07	(92.5%)	ERS985912	2.89E+07	(95.0%)	ERS985920	3.38E+07	(88.0%)	ERS985989	2.99E+07	(91.5%)	ERS985997	3.01E+07	(92.0%)	ERS986004	2.34E+07	(89.4%)	ERS985929	3.69E+07	(92.9%)	ERS986010	2.38E+07	(89.6%)	ERS985937
Germany (Cologne-Bonn) - 2	CB	GER2	male	TP121B	tp121	9	2.74E+07	(95.1%)	ERS985900	2.75E+07	(90.8%)	ERS985908	3.00E+07	(92.7%)	ERS985913	2.38E+07	(95.4%)	ERS985921	7.61E+07	(92.0%)	ERS986120	3.25E+07	(91.7%)	ERS985998	NA	NA	NA	1.97E+07	(89.2%)	ERS985930	3.14E+07	(91.5%)	ERS986011	2.13E+07	(89.4%)	ERS985938
Germany (Cologne-Bonn) - 2	CB	GER3	male	TP17-2	tp172	10	2.97E+07	(91.6%)	ERS985901	NA	NA	NA	2.90E+07	(93.6%)	ERS985914	2.68E+07	(94.5%)	ERS985922	5.33E+07	(94.8%)	ERS986121	3.33E+07	(91.4%)	ERS986000	5.09E+07	(96.1%)	ERS986122	2.16E+07	(92.6%)	ERS985931	3.23E+07	(92.5%)	ERS986012	2.28E+07	(89.0%)	ERS985940
Germany (Cologne-Bonn) - 2	CB	GER4	male	TP3-92	tp3a2	10	2.97E+07	(91.1%)	ERS985902	NA	NA	NA	3.17E+07	(92.5%)	ERS985915	2.61E+07	(95.2%)	ERS985923	3.73E+07	(90.8%)	ERS985992	3.20E+07	(90.8%)	ERS986001	3.72E+07	(92.8%)	ERS986006	2.08E+07	(92.6%)	ERS985932	3.50E+07	(92.3%)	ERS986013	2.51E+07	(89.6%)	ERS985941
Germany (Cologne-Bonn) - 2	CB	GER5	male	TP4a	tp4a	8	2.63E+07	(91.9%)	ERS985903	2.53E+07	(92.4%)	ERS985910	2.95E+07	(93.5%)	ERS985916	3.19E+07	(92.6%)	ERS985985	3.88E+07	(89.2%)	ERS985993	NA	NA	NA	3.75E+07	(93.0%)	ERS986007	2.11E+07	(92.3%)	ERS985933	2.55E+07	(93.7%)	ERS985925	2.04E+07	(89.4%)	ERS985942
Germany (Cologne-Bonn) - 2	CB	GER6	male	TP51D	tp51	9	3.01E+07	(91.4%)	ERS985904	NA	NA	NA	3.50E+07	(93.5%)	ERS985917	3.34E+07	(92.5%)	ERS985986	3.21E+07	(91.3%)	ERS985994	3.14E+07	(92.2%)	ERS986002	3.75E+07	(92.8%)	ERS986008	1.90E+07	(92.2%)	ERS985934	2.44E+07	(92.3%)	ERS985926	3.01E+07	(88.6%)	ERS985943
Germany (Cologne-Bonn) - 2	CB	GER7	male	TP7-10F1A2	tp710	8	2.85E+07	(92.0%)	ERS985905	NA	NA	NA	2.88E+07	(96.1%)	ERS985918	2.89E+07	(92.1%)	ERS985987	3.67E+07	(89.2%)	ERS985995	NA	NA	NA	3.03E+07	(93.0%)	ERS986009	2.16E+07	(92.8%)	ERS985935	2.08E+07	(92.0%)	ERS985927	2.17E+07	(86.8%)	ERS985944
Germany (Cologne-Bonn) - 2	CB	GER8	male	TP81B	tp81	9	2.89E+07	(91.6%)	ERS985906	2.62E+07	(90.7%)	ERS985911	2.92E+07	(96.0%)	ERS985919	3.31E+07	(90.0%)	ERS985988	3.47E+07	(89.0%)	ERS985996	3.02E+07	(90.3%)	ERS986003	2.83E+07	(96.0%)	ERS985924	1.79E+07	(92.8%)	ERS985936	2.15E+07	(88.9%)	ERS985928	2.28E+07	(86.5%)	ERS985999
Iran (Ahvaz) - 4	IR	IRA1b	male	(AH15)*	131*	10	1.92E+07	(93.1%)	ERS985949	2.34E+07	(89.9%)	ERS985958	2.53E+07	(91.2%)	ERS985966	2.10E+07	(93.6%)	ERS985975	2.77E+07	(90.7%)	ERS985983	3.17E+07	(95.2%)	ERS986022	3.63E+07	(95.5%)	ERS986031	3.29E+07	(94.6%)	ERS986043	2.75E+07	(94.5%)	ERS986036	2.75E+07	(90.7%)	ERS986052
Iran (Ahvaz) - 4	IR	IRA2b	male	(AH23)*	132*	10	2.29E+07	(92.4%)	ERS985950	2.41E+07	(91.0%)	ERS985959	2.46E+07	(92.1%)	ERS985967	1.99E+07	(93.8%)	ERS985976	3.30E+07	(92.5%)	ERS986015	2.98E+07	(95.1%)	ERS986023	2.42E+07	(94.9%)	ERS986032	3.82E+07	(95.1%)	ERS986045	2.23E+07	(94.4%)	ERS986037	2.26E+07	(90.5%)	ERS986053
Iran (Ahvaz) - 4	IR	IRA3b	male	(JR11)*	IR121*	9	2.01E+07	(90.9%)	ERS985951	2.18E+07	(91.4%)	ERS985960	2.43E+07	(94.0%)	ERS985968	2.30E+07	(93.0%)	ERS985977	3.63E+07	(89.1%)	ERS986016	3.12E+07	(94.7%)	ERS986024	NA	NA	NA	3.28E+07	(93.9%)	ERS986046	2.82E+07	(94.4%)	ERS985909	2.56E+07	(90.4%)	ERS986054
Iran (Ahvaz) - 4	IR	IRA4b	male	(JR15)*	IR122*	10	2.17E+07	(91.2%)	ERS985952	2.49E+07	(92.8%)	ERS985961	2.28E+07	(93.8%)	ERS985970	2.37E+07	(93.3%)	ERS985978	3.48E+07	(89.4%)	ERS986017	2.65E+07	(95.3%)	ERS986025	3.39E+07	(96.3%)	ERS986033	3.81E+07	(94.8%)	ERS986047	2.87E+07	(94.3%)	ERS986038	3.27E+07	(92.2%)	ERS986055
Iran (Ahvaz) - 4	IR	IRA5	male	JR2-F1C	02FC_1	10	2.05E+07	(92.6%)	ERS985945	2.00E+07	(92.2%)	ERS985953	2.63E+07	(93.9%)	ERS985962	2.20E+07	(93.5%)	ERS985971	2.16E+07	(89.1%)	ERS985979	2.64E+07	(94.9%)	ERS986018	3.38E+07	(94.9%)	ERS986026	3.30E+07	(94.3%)	ERS986039	2.87E+07	(93.2%)	ERS986034	2.63E+07	(91.0%)	ERS986048
Iran (Ahvaz) - 4	IR	IRA6	male	JR5-F1C	05F1C1	9	2.31E+07	(92.1%)	ERS985946	2.64E+07	(91.0%)	ERS985955	2.59E+07	(92.0%)	ERS985963	2.10E+07	(93.5%)	ERS985972	2.42E+07	(88.0%)	ERS985980	2.80E+07	(95.2%)	ERS986019	2.94E+07	(94.7%)	ERS986027	3.85E+07	(93.5%)	ERS986040	NA	NA	NA	3.08E+07	(90.2%)	ERS986049
Iran (Ahvaz) - 4	IR	IRA7	male	JR7-F1C	07F1C1	9	2.13E+07	(92.5%)	ERS985947	2.00E+07	(93.2%)	ERS985956	2.17E+07	(94.0%)	ERS985964	2.23E+07	(93.2%)	ERS985973	2.76E+07	(93.1%)	ERS985981	2.37E+07	(94.8%)	ERS986020	3.01E+07	(94.9%)	ERS986028	3.93E+07	(95.1%)	ERS986041	NA	NA	NA	2.79E+07	(90.8%)	ERS986050
Iran (Ahvaz) - 4	IR	IRA8	male	JR8-F1A	08F1_A	10	2.22E+07	(92.7%)	ERS985948	2.27E+07	(90.6%)	ERS985957	2.60E+07	(94.1%)	ERS985965	2.36E+07	(93.7%)	ERS985974	2.54E+07	(93.5%)	ERS985982	2.94E+07	(95.1%)	ERS986021	2.84E+07	(94.9%)	ERS986030	4.06E+07	(95.1%)	ERS986042	2.69E+07	(95.1%)	ERS986035	2.42E+07	(91.3%)	ERS986051
NA=tissue not available																																				

*these animals do not correspond to the animal used for genome sequencing, but are related to them. 131 and 132, as well as IR121 and IR122 are brother pairs.

**Table 3:** Summary of copy number variations

**Species**	**Population**	**Sample ID**	**CNVnator bin size**	**CNVnator average RD per bin+−StDev**	**number of detected CNVs**	**number of detected duplications**	**number of detected deletions**	**total bp Duplications** *	**total bp Deletions** †	**average CNV length (bp)**
Mus musculus domesticus	France (Massif Central)	14	200	56.18+−11.86	8,387	1,989	6,398	120,410,392	85,007,602	14,360
Mus musculus domesticus	France (Massif Central)	15B	200	53.72+−10.83	8,074	2,009	6,065	119,606,026	75,278,135	14,005
Mus musculus domesticus	France (Massif Central)	16B	200	56.95+−11.86	8,224	1,821	6,403	121,823,158	80,597,597	14,239
Mus musculus domesticus	France (Massif Central)	18B	200	55.97+−11.35	8,604	1,942	6,662	117,840,892	78,783,538	13,250
Mus musculus domesticus	France (Massif Central)	B2C	300	49.06+−10.31	7,893	4,494	3,399	208,235,138	42,812,601	14,287
Mus musculus domesticus	France (Massif Central)	C1	250	56.62+−11.67	6,512	1,977	4,535	131,564,896	66,289,715	16,128
Mus musculus domesticus	France (Massif Central)	E1	200	49.76+−10.27	8,337	2,470	5,867	141,568,026	72,799,133	13,789
Mus musculus domesticus	France (Massif Central)	F1B	200	51.967+−10.45	9,093	2,077	7,016	128,792,644	82,590,984	13,367
Mus musculus domesticus	Germany (Cologne-Bonn)	TP1	200	51.76+−10.48	9,237	2,511	6,726	131,918,198	91,211,874	14,041
Mus musculus domesticus	Germany (Cologne-Bonn)	TP121B	200	48.69+−10.12	8,060	2,536	5,524	129,474,342	87,053,076	15,765
Mus musculus domesticus	Germany (Cologne-Bonn)	TP17-2	200	53.33+−10.87	9,509	2,446	7,063	125,331,123	84,180,537	12,752
Mus musculus domesticus	Germany (Cologne-Bonn)	TP3-92	200	52.63+−10.55	8,673	2,386	6,287	119,924,724	73,493,913	12,593
Mus musculus domesticus	Germany (Cologne-Bonn)	TP4a	200	54.38+−11.02	9,498	2,560	6,938	153,808,543	82,965,662	13,275
Mus musculus domesticus	Germany (Cologne-Bonn)	TP51D	250	58.75+−12.23	7,834	2,123	5,711	133,889,956	68,350,789	13,939
Mus musculus domesticus	Germany (Cologne-Bonn)	TP7-10F1A2	200	44.06+−9.24	8,523	3,050	5,473	141,433,824	73,668,527	13,869
Mus musculus domesticus	Germany (Cologne-Bonn)	TP81B	200	49.31+−10.13	8,758	2,454	6,304	126,226,333	80,352,296	13,695
Mus musculus domesticus	Germany (Heligoland)	HG06	300	34.99+−8.40	4,418	1,065	3,353	80,826,282	39,943,747	14,447
Mus musculus domesticus	Germany (Heligoland)	HG08	300	45.04+−9.89	5,115	1,288	3,827	128,791,631	61,075,573	18,068
Mus musculus domesticus	Germany (Heligoland)	HG13	300	39.00+−9.43	4,296	1,040	3,256	80,934,382	39,571,844	14,890
Mus musculus domesticus	Iran (Ahvaz)	AH15	200	51.60+−10.34	9,415	1,966	7,449	134,664,482	97,134,751	14,460
Mus musculus domesticus	Iran (Ahvaz)	AH23	150	42.71+−9.26	11,863	3,885	7,978	167,287,101	78,048,722	11,040
Mus musculus domesticus	Iran (Ahvaz)	JR11	150	41.60+−8.76	13,033	3,161	9,872	166,512,111	91,877,878	10,631
Mus musculus domesticus	Iran (Ahvaz)	JR15	200	51.42+−10.56	9,553	2,223	7,330	139,340,206	90,151,070	13,832
Mus musculus domesticus	Iran (Ahvaz)	JR2-F1C	200	53.34+−10.78	10,799	2,737	8,062	155,155,231	89,942,938	12,549
Mus musculus domesticus	Iran (Ahvaz)	JR5-F1C	250	48.82+−10.97	8,885	5,434	3,451	305,615,567	51,740,299	19,427
Mus musculus domesticus	Iran (Ahvaz)	JR7-F1C	250	54.02+−11.20	7,496	2,859	4,637	186,709,744	69,846,363	16,807
Mus musculus domesticus	Iran (Ahvaz)	JR8-F1A	250	50.26+−10.49	6,794	1,974	4,820	157,434,749	74,771,930	18,028
Mus musculus musculus	Czech Republic (Studenec)	CR12	150	43.80+−8.86	27,563	2,509	25,054	112,116,722	101,183,846	4,764
Mus musculus musculus	Czech Republic (Studenec)	CR13	150	41.57+−8.60	26,485	2,313	24,172	110,524,786	100,033,329	4,857
Mus musculus musculus	Czech Republic (Studenec)	CR16	150	44.29+−9.03	26,588	2,547	24,041	128,365,321	110,932,459	5,473
Mus musculus musculus	Czech Republic (Studenec)	CR23	150	42.67+−9.34	25,310	2,243	23,067	110,705,202	101,170,533	5,095
Mus musculus musculus	Czech Republic (Studenec)	CR25	150	43.18+−9.05	25,665	2,274	23,391	112,031,543	97,872,009	4,848
Mus musculus musculus	Czech Republic (Studenec)	CR29	150	39.75+−8.32	26,023	2,277	23,746	104,768,374	101,404,004	4,946
Mus musculus musculus	Czech Republic (Studenec)	CR46	150	43.02+−8.80	26,134	2,530	23,604	140,999,291	107,372,796	5,457
Mus musculus musculus	Czech Republic (Studenec)	CR59	150	44.02+−9.42	25,528	2,272	23,256	107,909,196	102,440,094	5,070
Mus musculus musculus	Kazhakstan (Almaty)	AL1	150	41.32+−8.69	26,323	2,401	23,922	107,392,592	99,322,728	4,859
Mus musculus musculus	Kazhakstan (Almaty)	AL16	100	29.62+−7.05	32,759	4,080	28,679	146,326,242	116,025,321	4,787
Mus musculus musculus	Kazhakstan (Almaty)	AL19	150	42.47+−9.13	24,719	2,349	22,370	119,912,050	96,640,630	5,111
Mus musculus musculus	Kazhakstan (Almaty)	AL33	100	29.28+−6.90	35,421	3,443	31,978	105,579,838	110,556,022	3,915
Mus musculus musculus	Kazhakstan (Almaty)	AL38	150	43.90+−9.81	24,204	2,686	21,518	134,204,665	107,497,432	6,049
Mus musculus musculus	Kazhakstan (Almaty)	AL40	100	30.45+−6.86	35,975	3,509	32,466	110,320,912	107,338,334	3,787
Mus musculus musculus	Kazhakstan (Almaty)	AL41	100	30.16+−6.85	35,718	4,060	31,658	151,524,451	120,065,142	4,520
Mus musculus musculus	Kazhakstan (Almaty)	AL42	100	29.42+−6.64	35,700	3,539	32,161	112,866,183	178,368,939	5,821
Mus musculus musculus	Afghanistan (Mazar-e-Sharif)	396	400	86.97+−21.40	6,948	914	6,034	130,591,375	87,027,966	18,095
Mus musculus musculus	Afghanistan (Mazar-e-Sharif)	413	650	224.10+−54.67	4,182	566	3,616	103,073,558	52,147,184	20,917
Mus musculus musculus	Afghanistan (Mazar-e-Sharif)	416	750	174.34+−42.58	3,433	521	2,912	119,407,227	50,229,088	27,495
Mus musculus musculus	Afghanistan (Mazar-e-Sharif)	424	1,000	266.87+−63.44	2,487	497	1,990	128,701,356	38,560,010	36,180
Mus musculus musculus	Afghanistan (Mazar-e-Sharif)	435	1,000	329.37+−75.61	2,793	483	2,310	113,845,910	45,713,691	33,007
Mus musculus musculus	Afghanistan (Mazar-e-Sharif)	444	1,000	296.35+−71.09	2,507	479	2,028	137,096,002	40,478,972	39,479
Mus musculus castaneus	India (Himalaya)	H12	250	58.84+−13.53	16,306	1,820	14,486	404,919,636	142,150,764	19,224
Mus musculus castaneus	India (Himalaya)	H14	300	64.28+−15.50	12,395	1,144	11,251	68,755,079	101,402,549	10,031
Mus musculus castaneus	India (Himalaya)	H15	350	52.23+−12.50	9,883	981	8,902	52,107,722	89,674,048	11,271
Mus musculus castaneus	India (Himalaya)	H24	350	58.64+−14.17	9,800	981	8,819	55,307,963	89,606,581	11,379
Mus musculus castaneus	India (Himalaya)	H26	300	62.14+−15.33	11,252	1,323	9,929	83,964,222	92,145,571	10,675
Mus musculus castaneus	India (Himalaya)	H27	350	57.55+−13.56	10,325	1,039	9,286	61,747,428	92,045,614	11,196
Mus musculus castaneus	India (Himalaya)	H28	350	64.49+−15.56	10,087	1,290	8,797	431,408,968	117,023,503	28,917
Mus musculus castaneus	India (Himalaya)	H30	250	66.87+−16.26	14,572	1,505	13,067	66,990,211	107,996,933	9,140
Mus musculus castaneus	India (Himalaya)	H34	350	87.05+−20.72	9,841	1,336	8,505	404,724,739	110,588,695	28,444
Mus musculus castaneus	India (Himalaya)	H36‡	1,500	345.39+−79.26	2,127	248	1,879	52,759,236	47,563,121	32,637
Mus spretus	Spain (Madrid)	SP36	250	71.59+−17.33	20,718	1,079	19,639	61,984,254	147,878,111	8,213
Mus spretus	Spain (Madrid)	SP39	100	32.18+−7.75	51,832	2,820	49,012	97,981,334	226,304,088	4,926
Mus spretus	Spain (Madrid)	SP41	150	46.05+−9.85	41,977	1,745	40,232	78,690,640	200,024,818	5,249
Mus spretus	Spain (Madrid)	SP51	150	44.64+−10.00	39,620	1,555	38,065	65,460,386	148,332,035	4,201
Mus spretus	Spain (Madrid)	SP62	150	46.23+−10.21	40,977	1,576	39,401	62,811,481	156,532,699	4,251
Mus spretus	Spain (Madrid)	SP68	150	45.645+−9.97	39,751	1,706	38,045	83,305,283	195,303,505	5,551
Mus spretus	Spain (Madrid)	SP69	150	45.67+−10.71	34,947	1,544	33,403	75,886,262	192,900,647	6,076
Mus spretus	Spain (Madrid)	SP70	150	43.93+−9.40	41,394	1,681	39,713	85,765,326	205,565,887	5,499

*Number of bases for each CNV detected as duplication relative to the mm10 reference assembly was first multiplied by its reported copy number and the resulting numbers for all duplications were summed up to get total numbers presented in the table.

^†^Sum of number of bases detected as deletions relative to the mm10 reference assembly

^‡^ratio of the average read depth signal to its standard deviation was >5
