# Supplementary material for: Genomic resources for wild populations of the house mouse, Mus musculus and its close relative Mus spretus
Source: Sci Data. 2016 Sep 13;3:160075. doi: 10.1038/sdata.2016.75 (PMC5020872; doi:10.1038/sdata.2016.75)
Supplement: Supplementary Material [file sdata201675-s7.docx]

**Supplementary Material**

**Genomic resources for wild populations of the house mouse, *Mus musculus*, and its close relative *Mus spretus***

Bettina Harr, Emre Karakoc, Rafik Neme, Meike Teschke, Christine Pfeifle, Željka Pezer, Hiba Babiker, Miriam Linnenbrink, Inka Montero, Rick Scavetta, Mohammad Reza Abai, Marta Puente Molins, Mathias Schlegel, Rainer G. Ulrich, Janine Altmüller, Marek Franitza, Anna Büntge, Sven Künzel, Diethard Tautz

**Supplemental Text (within this document)**

**Text S1: Procedures for handling wild mice**

**Text S2: SNP and indel discovery**

**Supplemental Figures (within this document)**

**Figure S1: Fresh cage and enrichment material**

**Figure S2: Pictures of changing box**

**Figure S3: VSQR tranche plot**

**Figure S4: Regional variation in coverage of the genome for subspecies *M. m. domesticus***

**Figure S5: k-mer distributions (k=12) for three tissues (brain, testis, liver) in three randomly chosen individuals from each of the three *M. m. domesticus* populations**

**Supplemental Tables (provided as separate files)**

**Table S1: Summary of collection localities**

**Table S2: Breeding setup for *M. m. domesticus* mice**

**Table S3: RNAseq read mapping statistics**

**Table S4: Number of segregating sites and corresponding θ Watterson estimates for each population.**

**Table S5: Pairwise relatedness estimates for all possible pairs of individuals in the sample**

**Text S1: Procedures for handling wild mice**

**General considerations**

For wild mice environmental enrichment and material for nest building are placed in the cages (Figure S1) to dramatically reduce agitated stereotypic behaviour, like continuous somersaulting. When several mice are house in the same cage, each individual needs its own possibility for cover to avoid fights. The mice are kept in open cages (i.e. not individually ventilated) to ensure that they can interact with the environment. Specific-pathogen-free conditions are not required. Standard mouse chow is provided *ad libitum* (e.g. Altromin 1324 from ALTROMIN, 32791 Lage, Germany).

The maximum number of adult wild mice (20g bodyweight) in a standard type III cage (floor area 810 cm^2^) is five animals (for comparison: laboratory inbred strain mice are kept at a density of up to 13 adult mice on 810 cm^2^ floor area). Males can be kept at a maximum density of 2-3 individuals per cage until they reach 2-3 months of age, at which time they most likely will start fighting and need to be separated and housed individually.

Wild mice keep better hygiene than laboratory inbred strain mice, which implies that cages need to be changed less frequently (only about every two weeks). Since each cage change introduces stress to the animals, we try to minimize stress by partially transferring old nest material together with new nest material into the new cage.

**Figure S1: Fresh cage and enrichment material: (**A**)** Fleece paper, egg carton, wood wool, mouse igloo with a Fast-Trac running wheel (Plexx BV - http://plexx.eu/), small wood chips as bedding material. (B) Fully assembled cage with all its components including live wild mouse.

**Cage changing process**

Wild mice are much more agile than laboratory inbred strain mice. They readily jump out of open cages and are difficult to catch by hand. Since the mice jump out of their cages, as soon as the lid is removed, we use a special changing box (Figure S2) for changing cages. The changing box is custom built and is height-adjustable to allow optimal working conditions for animal care takers of different height. The adjusting mechanism does not use oil to allow the whole box to be washed in the rack washer. The bottom can be removed for separate regular cleaning. A cage intended for change is placed into the changing box before the lid is removed (Figure S2B). After removal of the lid, the mice usually leave their cage and are then caught in a transparent changing tube (Figure S2B). This tube is closed on one end and mice tend to enter it easily. They can then be lifted out and visually inspected for health and sex without extensive handling.


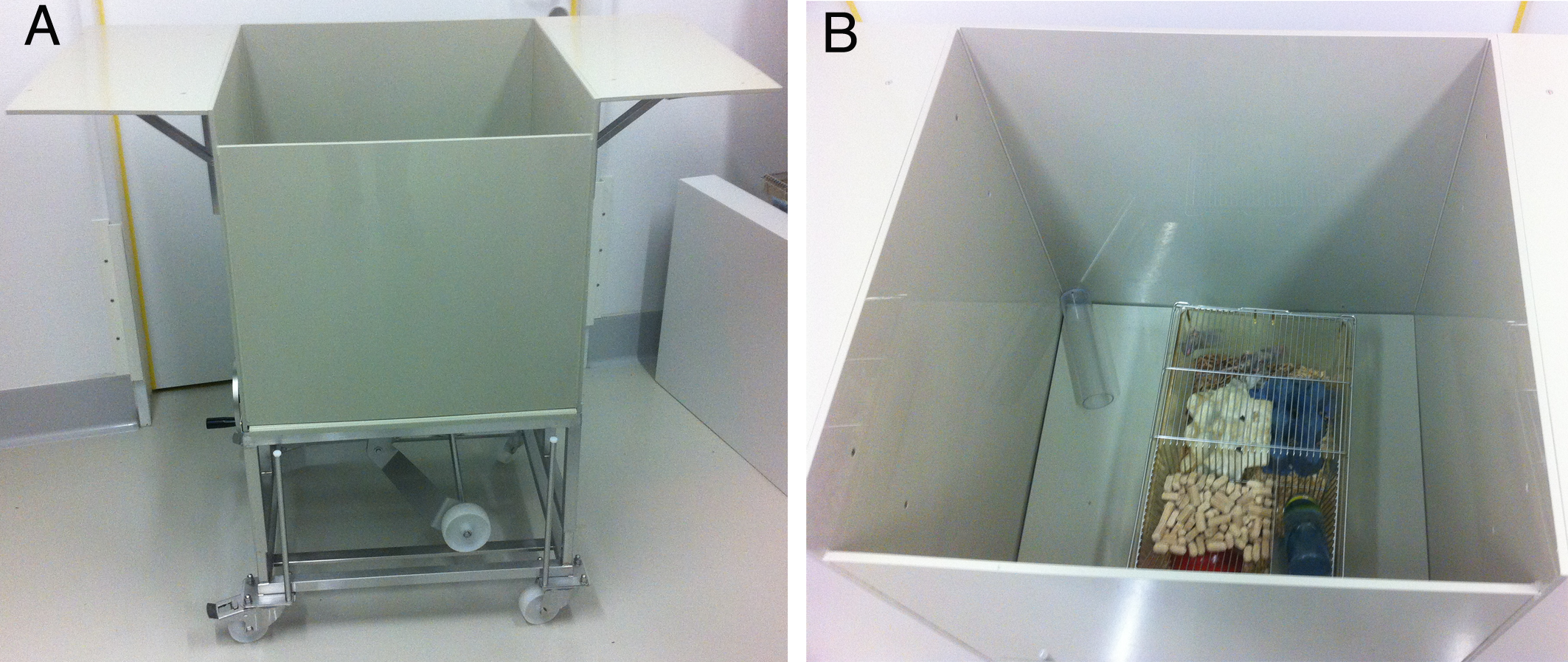


**Figure S2:** Pictures of changing box. (A) Complete box with device for height adjustment and side boards. (B) View into the box with a cage ready for change and a transparent changing tube for catching the mice.

**Setting up the breeding cage**

We always breed one female with one male as wild mice are territorial and the space of a typical type III cage (810 cm^2^) is not large enough for more than two mice in a breeding context. For some populations we have better breeding success when the female and the male are put together in a freshly prepared unsoiled cage. Wild females also seem to have some territorial requirements.

It is not possible to return sexually experienced males or females to their previous litter group. This would result in severe fights.

We do not determine the sex or number of pups of newly born litters as mothers are very sensitive towards disturbance and might kill their pups. Females are generally very easily disturbed during the lactation period. Therefore we keep the breeding room as quiet as possible with limited rotation of staff.

Litter size and sex of offspring is determined at weaning, which takes place roughly four weeks after birth (about 3-4 days later than for laboratory inbred strain mice). It is crucial to keep the mice in groups as long as possible for their social development.

**Health monitoring**

We use the laboratory strain Crl:CD1(ICR) from Charles River Laboratories (Wilmington, MA, USA) as bedding sentinels, i.e. externally sourced animals that are exposed to soiled bedding from the wild mice. This strain is known to be susceptible to most common pathogens. Every mouse room has one cage of sentinels. Direct contact sentinels cannot be used, since wild mice do not tolerate mice from inbred strains in their cage. At each cage change, soiled bedding from several wild mouse cage is collected, mixed and placed together with fresh bedding material into the cage housing sentinel mice. The mixed bedding rotates throughout all the cages in the room, so that after several cage changes the sentinel mice will have been exposed to bedding of each wild mouse cage in the room. Within the rack holding all mouse cages, the sentinel cage is last in the row. This position in the rack provides most exposure to particles circulating the whole room. The sentinels are sent to a commercial provider of health status analysis every six to eight months. The analysis includes a check of all viruses, bacteria, opportunists and parasites that are recommended by FELASA guidelines.

**Text S2: SNP and indel discovery**

We aligned the Illumina sequencing reads to the mouse reference genome (NCBI GRCm38) using the BWA-MEM aligner with the default options and paired-end mode. The reference sequence included the chromosome patches for mapping reads in *musculus*, *castaneus* and *spretus,* and mice from Heligoland*,* but excluded chromosome patches for mapping of *domesticus* reads from the German, French and Iranian population. Due to the elevated divergence between the mouse subspecies we compared the performance of the BWA-MEM with respect to other mappers such NGM (Sedlazeck 2013), which is designed for mapping to a reference with a high divergence. However BWA-MEM reported more mappings and proper paired-end mapping compared to NGM (data not shown).

Next, we used these alignments to discover SNPs and INDELs for each sample. We followed the general GATK version 3 best-practices pipeline. The first step of the variant discovery is the data clean-up. We used the Picard tools software to mark and filter the PCR duplicates in our alignments. Next, we used GATK to realign the indel-containing reads to the reference genome in order to correct the SNPs at the flanking regions for insertions and deletions. Then, we applied the base recalibration in order to reduce the false positives and false negatives during the variant calling phase.

These alignment files were then used by GATK’s HaplotypeCaller to detect SNPs and INDELs separately. The program first determines the regions of the genome that it needs to operate on, based on the presence of significant evidence for variation. For each active region, the program builds a De Brujin-like graph to reassemble the region and tries to identify the possible haplotypes present in the data. These haplotypes are then aligned to the reference using the optimal alignment method (Smith-Waterman) in order to identify the potential variant sites. Each read is aligned to the haplotypes using the PairHMM algorithm. This produces the likelihoods of the haplotypes given the reads. These likelihoods are used to estimate the likelihoods of the alleles for each potential variant site. For each potential variant site the Bayesian rule is applied using the likelihoods of the alleles to calculate the likelihoods of the genotypes. The most likely genotype is assigned to the sample. The genotype assignment is processed on each chromosome separately to safe time. Afterwards, chromosomes are merged for each sample. We excluded genotypes called on all chromosome patches from further analysis.

GenotypeGVCFs is used to merge the HaplotypeCaller generated .gvcf results into a joint .vcf file. These calls are then processed by GATK’s VariantFiltration to hard filter the SNPs. We used strict filtering criteria, as suggested by the GATK best practices guidelines (i.e., Fisher’s exact test for strand bias, root mean square of the mapping quality, u-based z-approximation from the Mann-Whitney Rank Sum Test for mapping qualities and u-based z-approximation from the Mann-Whitney Rank Sum Test for the distance from the end of the read), to determine a subset of good quality SNPs. The specific thresholds for each filter are given in the commands section below.

We also downloaded the dbSNP data from the Mouse Genome Consortium (Keane et al. 2011) (i.e. file “mgp.v5.merged.snps_all.dbSNP142.vcf” downloaded from <ftp://ftp-mouse.sanger.ac.uk/current_snps/> ) and filtered these SNPs for “PASS” SNPs. dbSNP “PASS” SNPs and in addition our hard-filtered high quality SNPS were then used as training sets to calibrate all our variants using GATK’s VariantRecalibrator. This tool performs the first pass in a two-stage process called VQSR; the second-pass is performed by the ApplyRecalibration tool. In brief, the first pass consists of creating a Gaussian mixture model by looking at the distribution of annotation values over a high quality subset of the input call set, and then scoring all input variants according to the model. The second pass consists of filtering variants based on score cut-offs identified in the first pass.

The purpose of the variant recalibrator VQSR is to assign a well-calibrated probability to each variant call in a call set. One can then create highly accurate call sets by filtering based on this single estimate for the accuracy of each call. We later used the probability of variant calls to get the final SNP results using a 90% threshold. Due to the absence of high quality INDEL reference dataset, we did not generate VQSR calls for indels.

Figure S3 shows the VSQR SNP tranche plot, which assists the user in deciding which level of stringency should be applied to the raw SNP calls, taking into consideration the transition/transversion ratio (ideally close to 2) and the number of true and false positive SNPs in the dataset (between 90% and 100%).

**Figure S3: VSQR tranche plot**

*Commands for Angsd relatedness and population structure analyses*

To calculate genotype likelihoods:

/usr/users/bharr/angsd/angsd -P 4 -b DOM_MUS_CAS_SPRET_HELGO_BAM_FILES -out DOM_MUS_CAS_SPRETUS_HEL_random_inbreeding -ref /usr/users/bharr/ILLUMINA/UCSC_REF_GENOME/mm10.fa -rf randomRegions10kb_1000loci -doCounts 1 -SNP_pval 2e-6 -minMapQ 30 -minQ 20 -minMaf 0.05 -GL 1 -doMajorMinor 4 -doMaf 1 -skipTriallelic 1 -doGlf 3

To calculate relatedness:

./ngsF/ngsF --n_ind 67 --glf DOM_MUS_CAS_SPRETUS_HEL_random_inbreeding.glf --n_sites 312689 --min_epsilon 1e-6 --out allNEW.approx_indF --approx_EM --seed 0 --init_values u -max_iters 500 --verbose 0

**Literature cited:**

Keane, T. M., L. Goodstadt, P. Danecek, M. A. White, K. Wong, B. Yalcin, A. Heger, A. Agam, G. Slater, M. Goodson, N. A. Furlotte, E. Eskin, C. Nellåker, H. Whitley, J. Cleak, D. Janowitz, P. Hernandez-Pliego, A. Edwards, T. G. Belgard, P. L. Oliver, R. E. McIntyre, A. Bhomra, J. Nicod, X. Gan, W. Yuan, L. van der Weyden, C. A. Steward, S. Bala, J. Stalker, R. Mott, R. Durbin, I. J. Jackson, A. Czechanski, J. A. Guerra-Assunção, L. R. Donahue, L. G. Reinholdt, B. A. Payseur, C. P. Ponting, E. Birney, J. Flint, and D. J. Adams. 2011. Mouse genomic variation and its effect on phenotypes and gene regulation. Nature 477:289–294.

Sedlazeck, F. J., P. Rescheneder, and A. von Haeseler. 2013. NextGenMap: fast and accurate read mapping in highly polymorphic genomes. *Bioinformatics* **29**:2790–2791.

**Commands mapping and genotype calling:**

Mapping:

bwa mem -M -t 12 -R @RG\tID:ERR019243\tLB:12\tPL:ILLUMINA\tSM:H30 /path_to_reference/mm10.fa /path_to_reads/ERR019243_1.fastq /path_to_reads/ERR019243_2.fastq | samtools view -bS - > H30.bam

samtools sort H30.bam H30.sorted

samtools index H30.sorted.bam

MarkDuplicates:

java -Xmx20g -jar /path_to_picard/MarkDuplicates.jar INPUT=/path_to_bam/H30.sorted.bam OUTPUT=/path_to_bam/H30.sorted.nodup.bam METRICS_FILE=/path_to_bam/H30.sorted.duplicate.metrics REMOVE_DUPLICATES=true ASSUME_SORTED=true TMP_DIR=/path_to_bam/tmp MAX_RECORDS_IN_RAM=500000 VALIDATION_STRINGENCY=LENIENT

samtools index H30.sorted.nodup.bam

IndelRealignment:

java -Xmx40g -d64 -jar /path_to_GATK/GenomeAnalysisTK.jar -T RealignerTargetCreator -nt 12 -I /path_to_bam/H30.sorted.nodup.bam
 -R /path_to_reference/mm10.fa -o /path_to_bam/H30.suspicious.indel.intervals.list

java -Xmx40g -d64 -Djava.io.tmpdir=/path_to_bam/H30.tmp -jar /path_to_GATK/GenomeAnalysisTK.jar -T IndelRealigner

-I /path_to_bam/H30.sorted.nodup.bam -R /path_to_reference/mm10.fa -targetIntervals /path_to_bam/H30.suspicious.indel.intervals.list -o /path_to_bam/H30.sorted.nodup.realigned.bam -compress 0 --maxReadsInMemory 1000000

BaseRecalibration:

java -Xmx20g -d64 -jar /path_to_GATK/GenomeAnalysisTK.jar -T BaseRecalibrator -nct 8 -I /path_to_bam/H30.sorted.nodup.realigned.bam
 -R /path_to_reference/mm10.fa -knownSites /path_to_dbsnp/ mgp.v5.merged.snps_all.dbSNP142_Sorted_Filtered_allChroms.recode.vcf -o /path_to_bam/ERR019152.recal.data.table

java -Xmx10g -d64 -jar /path_to_GATK/GenomeAnalysisTK.jar -T PrintReads -nct 8 -I /path_to_bam/H30.sorted.nodup.realigned.bam -
R /path_to_reference/mm10.fa -BQSR /path_to_bam/H30.recal.data.table -o /path_to_bam/H30.sorted.nodup.realigned.recalibrated.bam

Chromosomes separation:

samtools view -bh /path_to_bam/H30.sorted.nodup.realigned.recalibrated.bam chr1 > /path_to_chr/H30.chr1.bam

samtools index /path_to_chr/H30.chr1.bam

HaplotypeCaller:

java -Xmx5g -d64 -jar /path_to_GATK/GenomeAnalysisTK.jar -T HaplotypeCaller -nct 4 -R /path_to_reference/mm10.fa -I /path_to_chr/H30.chr1.bam --max_alternate_alleles 2 --emitRefConfidence GVCF -L chr1 --variant_index_type LINEAR --variant_index_parameter 128000 -o /path_to_gvcf/H30.chr1.vcf

Merge GVCFs:

java -cp /path_to_GATK/GenomeAnalysisTK.jar org.broadinstitute.gatk.tools.CatVariants -R /path_to_reference/mm10.fa -V /path_to_gvcf/H30.chr1.vcf -V /path_to_gvcf/H30.chr2.vcf … -V /path_to_gvcf/H30.chrY.vcf -out /path_to_gvcf/H30.all.vcf -assumeSorted

Joint Genotyping:

java -Xmx40g -d64 -jar /path_to_GATK/GenomeAnalysisTK.jar -T GenotypeGVCFs -R /path_to_reference/mm10.fa --variant H30.vcf --variant H15.vcf ... -o MouseGenomes.all.vcf

SNP filtering:

*Step 1: hard filtering of SNPs in own dataset*

java -jar /usr/product/bioinfo/GATK/3.1.1/GenomeAnalysisTK.jar -T VariantFiltration -R /usr/users/bharr/ILLUMINA/UCSC_REF_GENOME/mm10_full.fa -V MouseGenomes.all_FLAG_snps --filterExpression "QD < 2.0 || FS > 60.0 || MQ < 40.0 || MQRankSum < -12.5 || ReadPosRankSum < -8.0" --filterName "my_snp_filter" -o MouseGenomes.all_FILTER_snps.vcf

*Step 2: select only PASS SNPs from step 1*

vcftools --vcf MouseGenomes.all_FILTER_snps.vcf --remove-indels --remove-filtered-all --recode --recode-INFO-all --out MouseGenomes.all.filtered_snps_PASS.vcf

*Step 3: run the recalibration model, using the own (step 2) and Sanger (step 3) SNPs as training set*

java -jar /path_to_GATK/GenomeAnalysisTK.jar -T VariantRecalibrator -R /path_to_reference/mm10.fa -input MouseGenomes.all.vcf -resource:HARD,known=false,training=true,truth=true,prior=10.0 /path_to_gvcf/ MouseGenomes.all.filtered_snps_PASS.vcf -resource:sanger,known=false,training=true,truth=false,prior=12.0 /path_to_dbsnp/mgp.v3.snps.rsIDdbSNPv137.vcf -an DP -an QD -an FS -an MQRankSum -an ReadPosRankSum -mode SNP -tranche 100.0 -tranche 99.9 -tranche 99.0 -tranche 90.0 -recalFile MouseGenomes_new1_recalibrate_SNP.recal -tranchesFile MouseGenomes_new1_recalibrate_SNP.tranches -rscriptFile MouseGenomes_new1_recalibrate_SNP_plots.R

*Step 4: Apply recalibration*

java -jar path_to_GATK/GenomeAnalysisTK.jar -T ApplyRecalibration -R /path_to_reference/mm10.fa -input Domesticus.all.vcf -mode SNP --ts_filter_level 90 -recalFile MouseGenomes_new1_recalibrate_SNP.recal -tranchesFile MouseGenomes_new1_recalibrate_SNP.tranches -o MouseGenomes_all_90_recalibrated_snps_raw_indels.vcf

*Step 5: remove INDELS and/or filtered SNPs*

Vcftools can be used for the filtering of the final vcf file. The usage is similar to the step 2. For further options check the manual for vcftools.


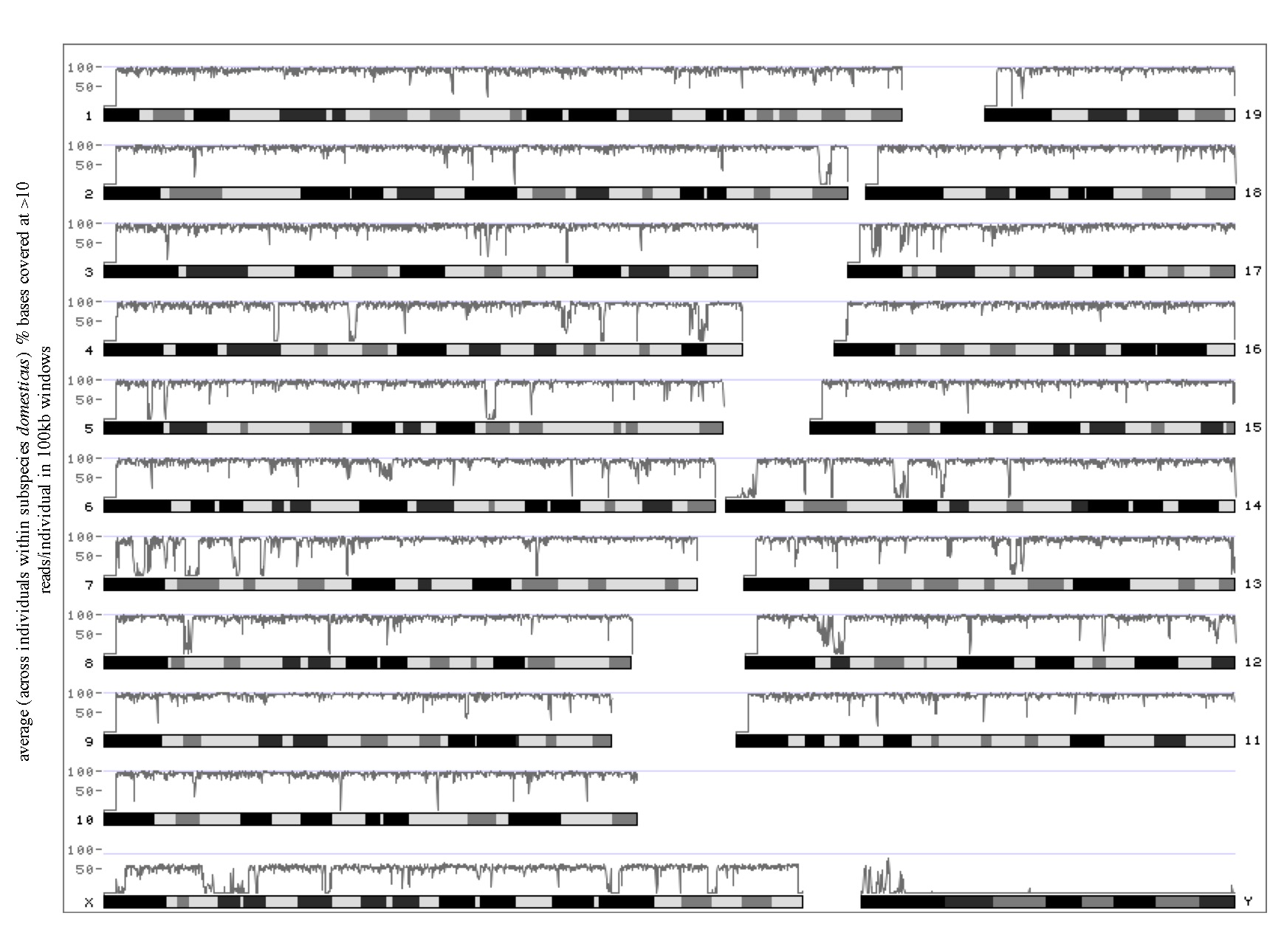


**Figure S4:** **Regional variation in coverage of the genome for subspecies *M. m. domesticus***

**
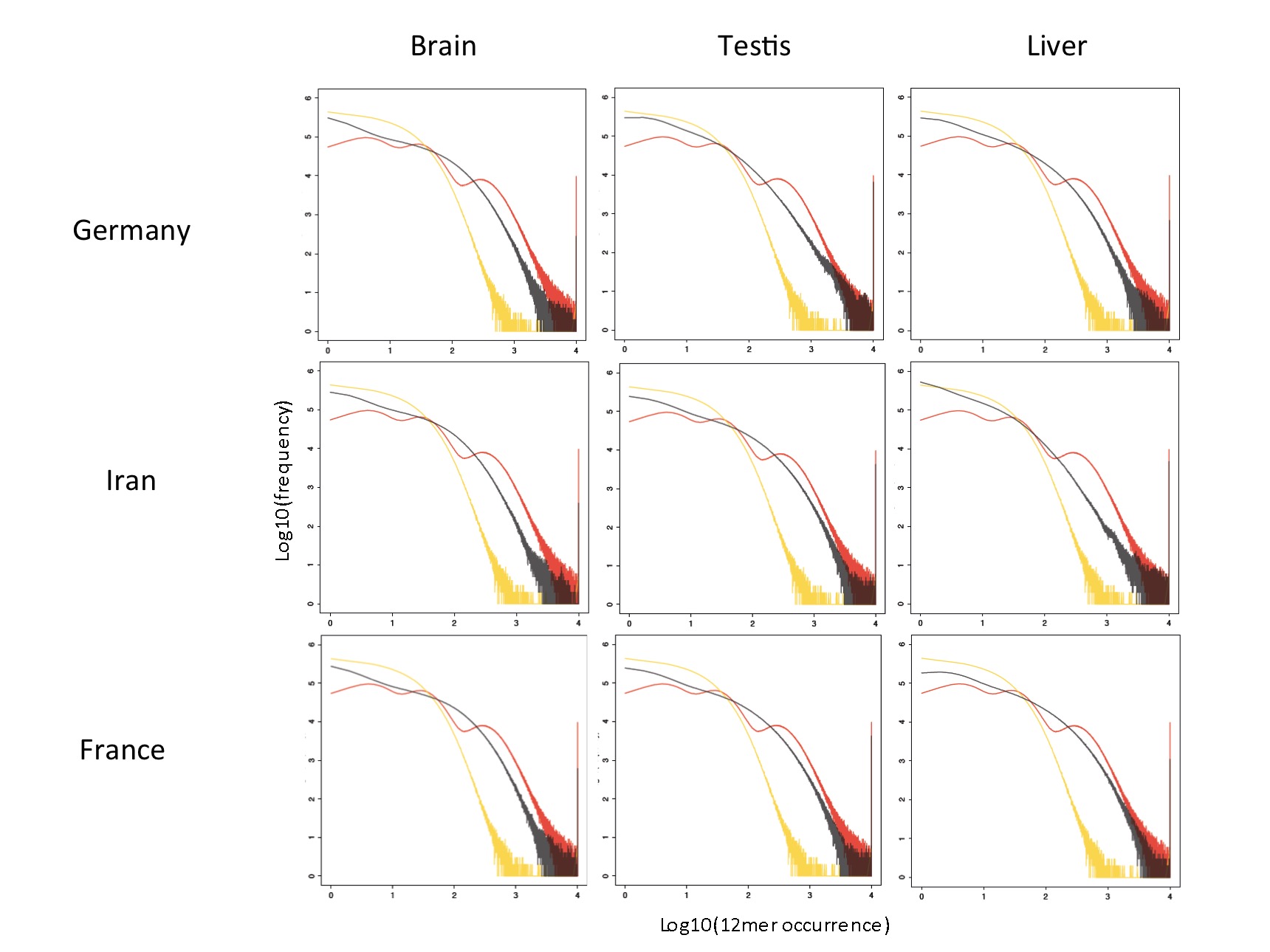
**

**Figure S5. K-mer distributions (k=12) for three tissues (brain, testis, liver) in three randomly chosen individuals from each of the three *M. m. domesticus* populations**

Red: k-mer distribution of genomic Sequence (mm10).

Yellow: k-mer distribution for known cDNS (ENSEMBL83)

Grey: k-mer distribution for RNAseq forward reads
